# RNAi-mediated suppression of three carotenoid-cleavage dioxygenase genes, *OsCCD1*, *4a*, and *4b*, increases carotenoid content in rice

**DOI:** 10.1093/jxb/ery300

**Published:** 2018-08-14

**Authors:** Mi Ran Ko, Mi-Hee Song, Jae Kwang Kim, Seung-A Baek, Min Kyoung You, Sun-Hyung Lim, Sun-Hwa Ha

**Affiliations:** 1 Department of Genetic Engineering and Graduate School of Biotechnology, College of Life Sciences, Kyung Hee University, Yongin, Republic of Korea; 2 College of Agriculture and Life Sciences, Chungnam National University, Daejeon, Republic of Korea; 3 Division of Life Sciences and Bio-Resource and Environmental Center, Incheon National University, Incheon, Republic of Korea; 4 National Academy of Agricultural Science, Rural Development Administration, Jeonju, Republic of Korea

**Keywords:** Carotenoid, carotenoid-cleavage dioxygenase, rice, *Oryza sativa*, RNA interference

## Abstract

Carotenoids of staple food crops have a high nutritional value as provitamin A components in the daily diet. To increase the levels of carotenoids, inhibition of carotenoid-cleavage dioxygenases (CCDs), which degrade carotenoids, has been considered as a promising target in crop biotechnology. In this study, suppression of the *OsCCD1*, *OsCCD4a*, and *OsCCD4b* genes using RNAi was verified in transgenic rice plants by quantitative RT-PCR and small RNA detection. Leaf carotenoids were significantly increased overall in *OsCCD4a*-RNAi lines of the T_1_ generation, and the highest accumulation of 1.3-fold relative to non-transgenic plants was found in a line of the T_2_ generation. The effects on seed carotenoids were determined via cross-fertilization between β-carotene-producing transgenic rice and one of two independent homozygous lines of *OsCCD1*-RNAi, *OsCCD4a*-RNAi, or *OsCCD4b*-RNAi. This showed that carotenoids were increased to a maximum of 1.4- and 1.6-fold in *OsCCD1*-RNAi and *OsCCD4a*-RNAi, respectively, with a different preference toward α-ring and β-ring carotenoids; levels could not be established in *OsCCD4b*-RNAi. In addition, the contents of four carotenoids decreased when *OsCCD1*, *OsCCD4a*, and *OsCCD4b* were overexpressed in *E. coli* strains accumulating phytoene, lycopene, β-carotene, and zeaxanthin. *OsCCD1* and *OsCCD4a* had a similar high carotenoid degrading activity, followed by *OsCCD4b* without substrate specificity. Overall, our results suggest that suppresing *OsCCD4a* activity may have potential as a tool for enhancing the carotenoid content of seed endosperms and leaves in rice.

## Teaser

Knock-down of the carotenoid-cleavage dioxygenase *OsCCD4a* in rice enhances the content of carotenoids in leaves and seeds.

## Introduction

Carotenoids, C_40_ terpenoid metabolites, play vital roles in plants as antenna pigments in photosynthesis, photo-protectants in the xanthophyll cycle, and precursors for the apocarotenoid hormones abscisic acid and strigolactone (Walter and Strack, 2011; Nisar *et al.*, 2015). In addition, dietary uptake of carotenoids benefits animals by providing health-enhancing properties for development and immune systems, and can also enhance body pigmentation that can confer a sexual selective advantage (Mayer *et al.*, 2008; Sommer and Vyas, 2012). In particular, humans require carotenoids as vitamin A precursors and antioxidants, and health problems are caused when they are deficient in the diet (Mayer *et al.*, 2008; Nisar *et al.*, 2015; Giuliano, 2017). As a consequence, biofortification of carotenoids has been a subject of ongoing interest in improvement of crop plants used for food and feed purposes, as summarized by Giuliano (2017). Diverse individual and combined techniques have been used to metabolically engineer the biosynthesis, degradation, sequestration, and stability of carotenoids by genetic modification (Zhai *et al.*, 2016; Giuliano, 2017). Strategies to block carotenoid catabolism have increased the contents of nutritionally valuable β-carotene and zeaxanthin via gene-silencing of the downstream steps of β-carotene hydroxylase (BCH) in potato tubers and zeaxanthin epoxidase (ZEP) in orange fruit and the competing branch step of lycopene ε-cyclase (LCY-e) in potato tubers (Römer *et al.*, 2002; Diretto *et al.*, 2006; Pons *et al.*, 2014). Wheat endosperm lacking carotenoids has been simultaneously engineered to produce carotenoids by ectopic expression of the bacterial phytoene synthase gene *CrtB* and to increase carotenoids by silencing the endogenous BCH-step gene *TaHyd* (Zeng *et al.*, 2015).

Similar gene-silencing strategies could be considered for blocking the degradation step of carotenoids into apocarotenoids, which are generated by the action of carotenoid-cleavage dioxygenases (CCDs) encoded by a multigene family consisting of two groups of 9-*cis*-epoxycarotenoid dioxygenases (NCEDs) and CCDs (Giuliano *et al.*, 2003; Auldridge *et al.*, 2006; Walter and Strack, 2011; Frusciante *et al.*, 2014; Hou *et al.*, 2016). Homologous genes belonging to the CCD group from diverse plant sources include CCD1, CCD2, CCD4, CCD7, and CCD8. The negative correlation found between the expression of either CCD1 or CCD4 and carotenoid accumulation suggests that they have a role in modulating carotenoid content in diverse plant species and organs, including chrysanthemum petals, potato tubers, orchid flowers, peach fruit, and Arabidopsis seeds (Ohmiya *et al.*, 2006; Campbell *et al.*, 2010; Chiou *et al.*, 2010; Brandi *et al.*, 2011, Gonzalez-Jorge *et al.*, 2013). Turning to staple crop plants, a strong up-regulation of *ZmCCD1* transcripts in the white endosperm of maize correlates with a lack of carotenoid accumulation (Vallabhaneni *et al.*, 2010). In contrast, no effect on carotenoid accumulation was reported in Golden rice when *OsCCD1* was expressed in both sense and antisense directions, suggesting that OsCCD1 specifically degrades apocarotenoids rather than carotenoids (Ilg *et al.*, 2010); however, transcript levels of *OsCCD1* in antisense plants were not discriminatively examined from those of *OsCCD1* in sense plants, which increased in the range of 50- to 240-fold. Recently, other *CCD* candidates have been used to boost carotenoid accumulation in common rice plants with white endosperm that fundamentally lacks carotenoid accumulation. Yang *et al.* (2017) found that *OsCCD4a* and *OsCCD4b* had no effects on carotenoid accumulation when they were knocked out using the RNA-guided targeted genome-editing technology known as CRISPR-CAS9. However, their abilities to enhance carotenoid content might still be possible in seeds of carotenoid-accumulating rice plants with a golden color phenotype.

In the current study, in order to fully elucidate the effects of *OsCCD1*, *OsCCD4a*, and *OsCCD4b* on carotenoid content, three genes were suppressed by RNAi in common rice plants, which were then conventionally cross-bred with a *stPAC* rice strain with a golden hue—this strain having previously been engineered to accumulate carotenoids in the endosperm (Jeong *et al.*, 2017). The *in vitro* carotenoid-cleavage activities of *OsCCD1*, *OsCCD4a*, and *OsCCD4b* were assessed in four carotenoid-accumulating *E. coli* strains, and carotenoid content was enhanced *in planta* by blocking carotenoid degradation by *OsCCD4a* in the leaves of common rice and by *OsCCD1* and *OsCCD4a* in seeds of carotenoid-accumulating rice.

## Materials and methods

### Rice plant material

Seeds of *japonica*-type Korean rice (*Oryza sativa* L. cv. Ilmi) were obtained from the National Institute of Crop Science, Rural Development Administration, Jeonju, Republic of Korea, and were used to analyse endogenous gene expression, to amplify the genes of interest, and to perform plant transformations. Seeds sterilized with 70% ethanol and 2% sodium hypochlorite were germinated on Murashige–Skoog agar medium for 1 week in a growth chamber, transplanted into soil, and were then grown in a greenhouse with natural light under a 16/8 h light/dark cycle at 28 °C. Transgenic plants of the T_0_ generation were acclimatized in a growth chamber and grown in a greenhouse until maturity. Rice plants were grown in the field during the summer season after T_1_ generation and interbreeding. Their seeds were harvested at 40 d after flowering (DAF), and the endosperm color was visually inspected after removing husks using a TR-200 Electromotion rice husker (Kett, Tokyo, Japan) followed by polishing with a Pearlest Polisher (Kett).

### Analysis of endogenous expression and sequence information for plant *CCD* genes

Samples harvested from diverse tissues of rice plants at different developmental stages were used for total RNA extraction with a RNeasy Mini Kit (Qiagen) according to the manufacturer’s instructions. The cDNA synthesized from 1 μg of total RNA was used for quantitative real-time (qRT)-PCR in a final volume of 20 µl, which included 0.25 µl of cDNA, 10 µl Thunderbird^TM^ SYBR qPCR Mix (Toyobo, Osaka, Japan), and 200 nM of each gene-specific primer pair(see Supplementary Table S1 at *JXB* online), on a CFX Connect^TM^ Real-Time System (Bio-Rad Laboratories) under PCR conditions of 1 min at 95 °C and 40 cycles of 15 s at 95 °C and 1 min at 60 °C. After the specificity of the PCR amplification was verified with melting-curve analysis (60 °C to 95 °C), threshold cycles (*C*t; the cycle at which the increase in fluorescence exceeded the threshold setting) were automatically determined. The expression level was analysed using CFX manager™ v.2.1 (Bio-Rad Laboratories) using the *OsUbi5* gene (Os01g22490) as a reference.

The sequences and structures of the plant *CCD* genes were searched from the database of the National Center for Biotechnology Information (NCBI;https://ncbi.nlm.nih.gov/) and the Knowledge-based *Oryza* Molecular Biological Encyclopedia (KOME). Protein sequence alignment was performed using the ClustalW algorithm of MegAlign in the DNASTAR program (https://dnastar.com/), and transit peptide sequence for chloroplast targeting was predicted using the ChloroP algorithm version 1.1 (http://www.cbs.dtu.dk/services/ChloroP/).

### Vector construction, transformation, and cross-fertilization of rice plants

*OsCCD1* (Os12g44310), *OsCCD4a* (Os02g47510), and *OsCCD4b* (Os01g22490) gene fragments were amplified from rice leaf cDNAs using gene-specific primer pairs in the first PCR of a Gateway cloning strategy. After performing a second PCR using the common *attB* primer pair, three products were finally introduced into a *pANDA-β* vector (Miki and Shimamoto, 2004) to mediate the RNAi to suppress gene expression via recombination by Gateway cloning (Invitrogen). All primer pairs are listed in Supplementary Table S1.

The three final RNAi vectors were transformed into *E. coli* DH5α and then into *Agrobacterium* LBA4404 and co-cultivated with embryogenic calli induced from mature rice seeds (*O. sativa* cv. Iimi). Green calli generated on selection medium containing phosphinothricin (4 mg l^–1^) and cefotaxime (500 mg l^–1^) were regenerated into transgenic plants via shooting and rooting procedures.

To cross-breed the *OsCCD1*-RNAi, *OsCCD4a*-RNAi, and *OsCCD4b*-RNAi lines with the *stPAC* rice strain that displays a golden color due to carotenoid production in the endosperm (Jeong *et al.*, 2017), conventional interbreeding during field growth in the summer season was performed at the T_3_ generation between two independent homozygous lines of the three RNAi transgenic plants as female parents and the homozygous *stPAC 25* with a single intact copy of the transgene as the male parent. The resultant F_1_ seeds were self-pollinated for two more generations in the field to obtain homozygosity for the transgene traits of both parents.

### Molecular analysis of transgenic rice plants

The genomic DNA of transgenic rice plants was extracted from leaf tissues of the T_0_ and F_3_ generations using a modification of the CTAB extraction method. To confirm the introduction of the transgene, genomic DNA PCRs were performed with the transgene-specific primer pair and the common *attB* primer pair of the Gateway binary vector (Supplementary Table S1). For Southern blot analysis, 20 μg samples of each genomic DNA from leaf tissues of the T_0_ generation were used for *EcoR*I digestion and then separated on 1% agarose gel to make the Hybond N+ nylon membrane blot (Amersham Pharmacia). The membrane was hybridized with the *Npt*II gene probe amplified with a specific primer pair (5′-GAAGGGACTGGCTGCTATTG-3′ and 5′-AATATCACGGGTAGCCAACG-3′) using a PCR DIG Probe Synthesis Kit (Roche). Hybridization and detection were carried out with a DIG High Prime DNA Labeling and Detection Starter Kit II (Roche). The signal bands were developed after exposure to Lumi-Film Chemiluminescent Detection film (Roche) for 12 h and photographed in an Image Analyzer (FLA 3000, Fujifilm).

To verify transgene homozygosity in the T_3_ and F_3_ generations, TaqMan PCRs were performed to detect *Bar* as a transgene and the α-tubulin gene as an internal reference in the rice genome. The *Bar* assay included a primer pair (5′-GCACGCAACGCCTACGA-3′ and 5′-CACCAGCGGACGGGA-3′) and a customized probe (5′-CCGTGTACGTCTCCC-3′) labeled with a 6-carboxyfluorescein (6-FAM) fluorescent reporter dye. The α-tubulin assay included a fluorescent reporter dye VIC-labeled probe that is commercially available (Assay ID: Os03643486_s1; Applied Biosystems). The PCR reaction was performed using a TaqMan Gene Expression Master Mix (Applied Biosystems) on a CFX Connect^TM^ Real-time System (Bio-Rad Laboratories) with one cycle of 95 °C for 10 min and 40 cycles of 94 °C for 30 s, 58 °C for 40 s, and 68 °C for 1 min, as described by Song *et al.* (2016). The copy number of the T_3_ leaf genomic DNA of *stPAC 25* rice was calculated to have a value of 1 and verified to have a homozygous single-copy reference of the *Bar* transgene (Jeong *et al.*, 2017) using CFX Manager Software (Bio-Rad Laboratories).

The qRT-PCRs of transgenic plants were performed with total RNAs extracted from 78-d-old leaf tissues of T_1_ plants according to the same PCR conditions and primer pairs that were used for analysis of endogenous gene expression described above. For detection of small interfering RNAs (siRNAs), 1-month-old leaf tissue of the T_2_ plant generation was used for the consecutive extraction of total RNAs and small RNAs according to the method of Martin *et al.* (2005). RNA samples (8 μg) were separated on a 15% (w/v) denaturing polyacrylamide gel (86 mm wide, 68 mm long, 1.5 mm thick) containing 7 M urea and 1× Tris-Borate-EDTA (TBE) buffer (pH 8.0) through a pre-run at 40 V for 1 h and a run at 150 V for 1 h. The RNAs were transferred onto a Hybond-N^+^ nylon membrane (GE Healthcare) using a Semi-Dry Transfer Unit (Bio-Rad Laboratories), UV cross-linked, and hybridized in Rapid-hyb^TM^ buffer (GE Healthcare). Gene-specific probes for *OsCCD1*, *4a*, and *4b* were prepared as ^32^P-dCTP-labeled PCR fragments using a Random Prime Labeling System Rediprime II^TM^ (GE Healthcare). The membrane was exposed to a Phosphor Imaging Screen (GE Healthcare) for 3 d and then visualized using the Molecular Imager FX System (Bio-Rad).

### Carotenoid and chlorophyll analyses of transgenic rice plants

Carotenoids were extracted from the same T_1_ and T_2_ leaf tissues used for qRT-PCR and siRNA detection, and from mature F_4_ seeds (40 DAF) that were harvested from F_3_ plants for TaqMan PCRs and genomic PCRs as described in two previous reports for leaf and seed carotenoids, respectively (Song *et al.*, 2016; Jeong *et al.*, 2017). For HPLC analysis, the extracted samples were prepared by dissolving in 50:50 (v/v) dichloromethane/methanol after the addition of β-apo-8′-carotenal (0.05 ml, 25 μg ml^–1^, Sigma-Aldrich) as an internal standard, extracted as separated layers with hexan (1.5 ml), and then desiccated under liquid nitrogen. Carotenoids were then separated in an YMC ODS C-30 column (3 µm, 4.6 × 250 mm; YMC Europe) using an Agilent 1100 Series HPLC system equipped with a photodiode array detector under elution conditions as described previously (Song *et al.*, 2016). For quantitative analysis, the HPLC chromatograms generated at 450 nm were used for determination of the peak areas relative to those of the calibration curves, which plotted at four different concentrations of individual carotenoid standards based on the peak area ratios with β-apo-8′-carotenal. Carotenoid standards were purchased from CaroteNature (Lupsingen, Switzerland) and included α-carotene (β,ε-carotene), 13*Z*-β-carotene, (all-*E*)-β-carotene, 9*Z*-β-carotene, lutein (β,ε-carotene-diol), zeaxanthin (β,β-carotene-diol), β-cryptoxanthin (β,β-caroten-ol), antheraxanthin (dihydro-epoxy-β,β-carotene-diol), and violaxanthin (diepoxy-tetrahydro-β-carotene-diol). The contents of 13*Z*-β-carotene, (all-*E*)-β-carotene, and 9*Z*-β-carotene were combined as that of β-carotene.

Total chlorophylls were extracted from the same T_2_ leaf tissues for both siRNA detection and carotenoid analysis and quantified as chlorophyll *a* (666 nm) and *b* (653 nm) in an Optizen POP spectrophotometer (Mecasys Company, Daejeon, Republic of Korea) as previously described (Song *et al.*, 2016).

For statistical analysis, all individual samples were analysed for metabolite quantification in triplicate. The relative differences between groups to non-transgenic plants were determined using a one-tailed Student’s *t*-test.

### Vector construction, transformation, and carotenoid analysis for carotenoid-accumulating *E. coli* strains

Full-length genes that included the open reading frames of *OsCCD1* (Os12g44310), *OsCCD4a* (Os02g47510), and *OsCCD4b* (Os01g22490) were amplified from rice leaf cDNA using gene-specific primer pairs (Supplementary Table S2). Common *attB* primer pairs were used to create three second PCR products that were introduced into a *pDEST15* vector via the Gateway cloning procedure (Invitrogen). The resultant bacterial expression vectors *pOsCCD1*, *pOsCCD4a*, and *pOsCCD4b* (see Supplementary Fig. S5) were transformed into *E. coli* strain BL21-AI^TM^ (Invitrogen), which has a tightly regulated L-arabinose-inducible T7 RNA polymerase system.

To provide phytoene, lycopene, β-carotene, and zeaxanthin as substrates for the carotenoid-cleavage action of rice CCDs, four *E. coli* strains harboring either *pPHYT*, *pLYC*, *pBETA*, or *pZEAX* (see Supplementary Fig. S5) were kindly provided by Professor DellaPenna’s group at Michigan State University (Cunningham *et al.*, 1996). After plasmid extraction using a Plasmid DNA Purification Kit (Macherey-Nagel), *pPHYT*, *pLYC*, *pBETA*, and *pZEAX* were individually transformed into competent BL21-AI *E. coli* cells harboring either *pOsCCD1*, *pOsCCD4a*, or *pOsCCD4b* vectors, and their transformations were verified by colony PCR with specific primer pairs (Supplementary Table S2) under PCR conditions of one cycle at 95 °C for 5 min and 25 cycles at 98 °C for 10 s, 65 °C for 30 s, and 72 °C for 2 min.

*E. coli* strains in a log-growth phase of OD_600nm_ 0.5 were treated with 0.2% (w/v) arabinose to induce the overexpression of *OsCCD1*, *4a*, and *4b*. After overnight incubation with shaking followed by centrifugation, pellet colors of *E. coli* cells were visually inspected, and the carotenoid content was analysed by HPLC. In brief, wet *E. coli* cells were pelleted at 14000 *g* for 40 s at 4 °C, suspended in 300 µl of acetone and 0.2 ml of *β*-apo-8′-carotenal as an internal standard, vortex-mixed for 10 s, and sonicated for 5 min. After incubation at 55 °C for 15 min in the dark, vortexing for 10 s, and centrifugation at 14000 *g* for 10 min at 4 °C, the supernatants were transferred to a new tube for carotenoid analysis using HPLC by the same method described above for rice leaves and seeds.

## Results

### Expression of *OsCCD1, OsCCD4a*, and *OsCCD4b* among various tissues and developmental stages

**Fig. 1.**
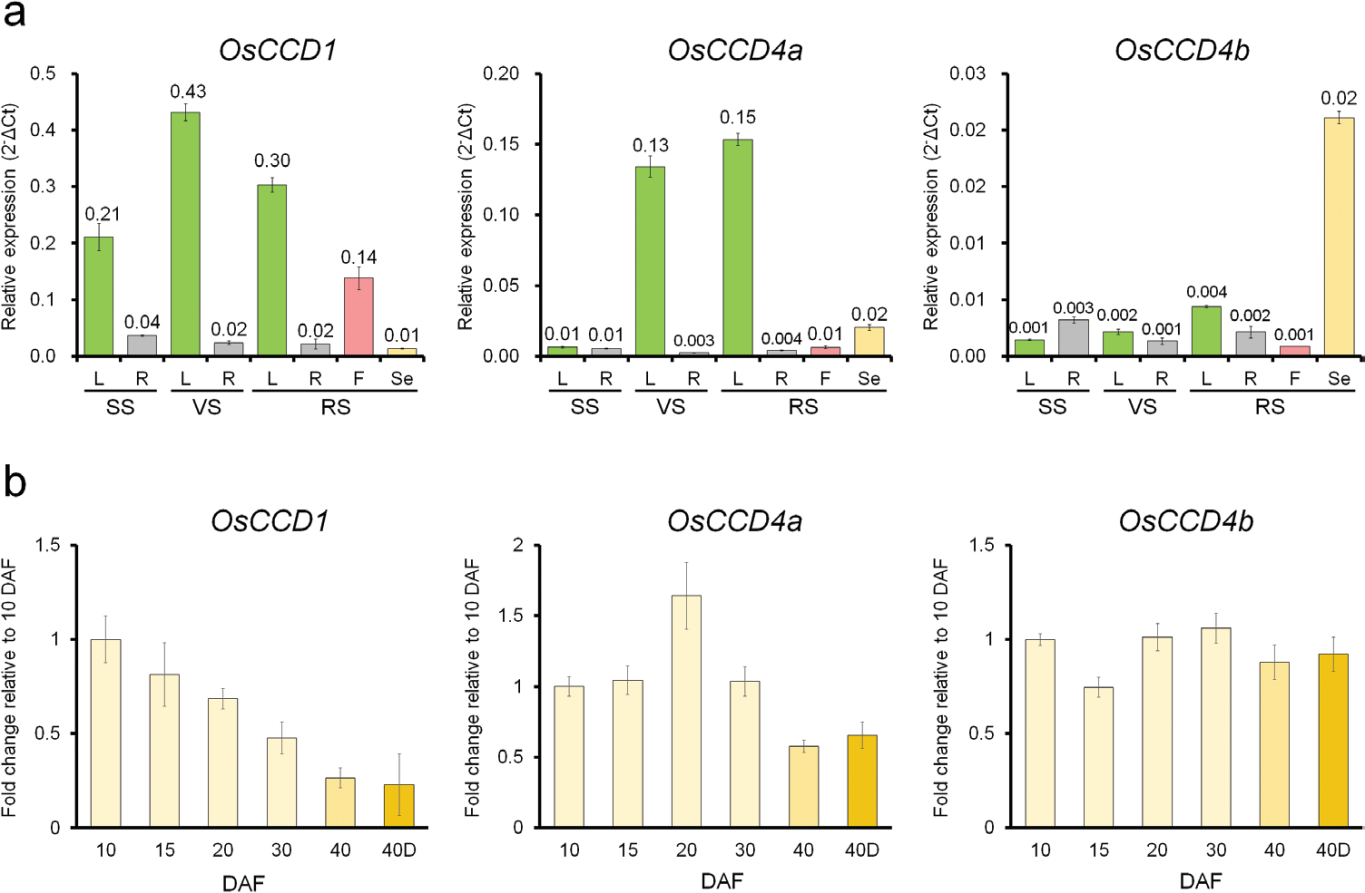
Endogenous transcript levels of *OsCCD1*, *OsCCD4a*, and *OsCCD4b* in various tissues at different developmental stages in rice. (a) The relative expression levels of the genes quantified by qRT-PCRs in leaves (L), roots (R), flowers (F), and seeds (Se) harvested at 40 d after flowering (DAF), at the seedling stage (SS), vegetative stage (VS), and reproductive stage (RS). (b) The relative expression levels of the genes quantified by qRT-PCRs during the development of seeds at 10, 15, 20, 30, and 40 DAF and at an additional stage that was seeds at 40 DAF that had been desiccated for 1 week (40D). The mean (±SE) *C*t values of triplicate measurements were used to calculate the expression of the target gene with normalization to an internal control (*OsUbi5*) using the Δ*C*t (a) and ΔΔ*C*t (b) methods.

To profile the expression patterns of *OsCCD1*, *OsCCD4a*, and *OsCCD4b*, rice RNAs from various tissues (leaves, roots, florets, and seeds) at different development stages (seedling, vegetative, and reproductive) were examined by qRT-PCR. The results showed that the expression of *OsCCD1* was ubiquitous, with higher levels in leaves than in other tissues (Fig. 1a). There was also higher expression of *OsCCD4a* in leaves after the seedling stage, but at lower levels relative to *OsCCD1*. The levels of expression of *OsCCD4b* were generally very low in all samples except for seeds at 40 DAF, which showed similar expression to *OsCCD4a* and slightly higher expression than *OsCCD1*.

The expression of *OsCCD1*, *OsCCD4a*, and *OsCCD4b* was examined during five stages of seed development at 10, 15, 20, 30, and 40 DAF and at an additional stage that was seeds at 40 DAF that had been desiccated for 1 week (Fig. 1b). Interestingly, the expression of the three genes displayed different patterns as seeds matured. *OsCCD1* was most highly expressed at the earliest stage (10 DAF) and then gradually decreased. The expression level of *OsCCD4a* was highest at the middle stage (20 DAF), whilst *OsCCD4b* was more or less the same across all stages. Desiccation did not affect the expression of the three genes after maturation.

### Molecular characteristics of *OsCCD1*, *OsCCD4a*, and *OsCCD4b* based on protein sequence analysis

A phylogenetic analysis of CCD1 and CCD4 protein sequences among plants was performed using the sequence database from the NCBI and the ClustalW algorithm of MegAlign in DNASTAR (Supplementary Fig. S1a). Both the CCD1 and CCD4 groups, including an OsCCD1 and two OsCCD4 proteins, were positively distinguished among plants. One OsCCD1 and two OsCCD4 proteins had a closer relationship with those of the monocotyledonous plants *Zea mays* (ZmCCD1) and *Crocus sativus* (CsCCD1 and CsCCD4a, CsCCD4b, CsCCD4c, and CsZCD) than with other proteins of dicotyledonous plants.

**Fig. 2.**
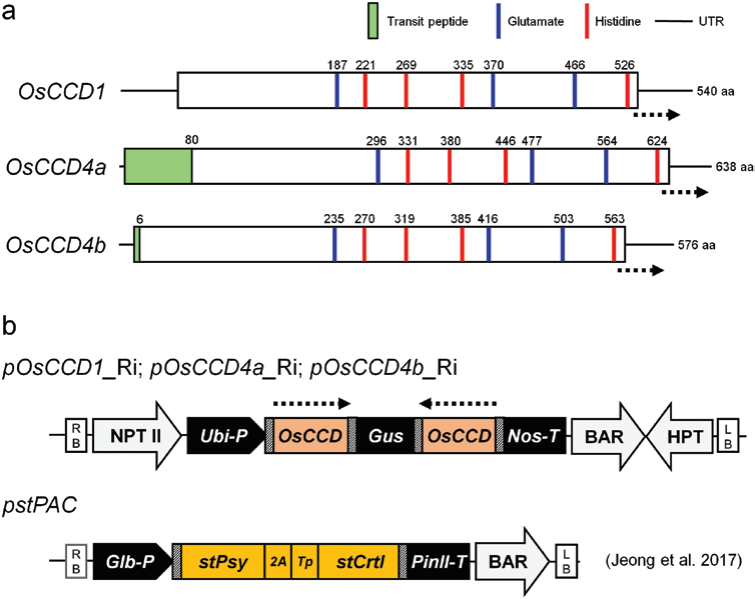
Schematic representation of rice *OsCCD1*, *OsCCD4a*, and *OsCCD4b* gene structures and binary vectors used in this study. (a) The conserved position and numbers of key amino acids known for enzymatic action of CCDs and transit peptide regions predicted using the ChloroP program. (b) Diagram of RNAi-mediated suppression vectors of the three genes. The *pstPAC* vector used to endow the endosperm of *stPAC* rice with a carotenoid-intensifying golden color trait was developed in a previous study (Jeong *et al.*, 2017) and is presented here because *stPAC* was used as a male parent in conventional interbreeding in this study. Dotted arrows in (a, b) indicate the gene region used in vector construction for RNAi-mediated suppression. In (b) the bacterial attachment *attB* sites needed for Gateway cloning are marked with hatched boxes. RB, right border; LB, left border; NPT II, neomycin-resistant gene cassette; BAR, bialaphos-resistant gene cassette; HPT, hygromycin-resistant gene cassette; *Ubi-P*, maize ubiquitin 1 promoter and 1st intron including splicing acceptor site; *Nos-T*, 3′-region from the nopaline synthase gene; *Glb-P*, rice globulin promoter; *PinII-T*, the 3′-region of the potato proteinase inhibitor II gene; *stPsy*, rice codon-optimized synthetic gene encoding *Capsicum* phytoene synthase (PSY); *2A*, rice codon-optimized foot-and-mouth disease virus 2A peptide; *Tp*, transit peptide of rice Rubisco small subunit; *stCrtI*, rice codon-optimized synthetic gene encoding bacterial desaturase (CRTI).

The protein structures of OsCCD1, CsCCD4a, and CsCCD4b were compared via sequence alignment among CCDs, including two Arabidopsis proteins (AtCCD1 and 4), a VP14 (*Zea mays* NCED), and three rice NCED proteins (OsNCED1, OsNCED2, and OsNCED3) (Supplementary Fig. S1b). The OsCCDs showed highly conserved characteristics as a CCD family, and these included NCED, a small CCD group that was distinct from NCED, and a distinct subgroup between CCD1 and CCD4 (Supplementary Fig. S1b). Key amino acid residues, including three glutamates and four histidines for CCD enzyme function, were also highly conserved in position and numbers (Supplementary Fig. S1b, Fig. 2a). The prediction of transit peptide (TP) sequences of OsCCDs using the ChloroP program indicated that OsCCD1 may not have a TP sequence, whereas two OsCCD4s have TP sequences of 80 and 6 amino acids (Fig. 2a), implying localization into the cytosol and chloroplasts, respectively.

### Generation of RNAi-mediated suppression lines for *OsCCD1, OsCCD4a*, and *OsCCD4b* in rice plants

For gene-specific suppression of *OsCCD1*, *OsCCD4a*, and *OsCCD4b* in rice plants, three RNAi-mediated vectors were constructed using cDNA sequences that included the C-terminal and 3′-untranslated regions (Fig. 2b). Ten transgenic plants for *OsCCD1*-RNAi, nine for *OsCCD4a*-RNAi, and nine for *OsCCD4b*-RNAi were generated, and their T-DNA integrations into the rice genome were confirmed by transgene-specific PCRs displaying 258-, 177-, and 226-bp amplicons, respectively (Supplementary Fig. S2a). After Southern blot analysis using an NPT II probe within a vector backbone (Supplementary Fig. S2b), three independent lines with an RNAi construct for *OsCCD1*, *OsCCD4a*, and *OsCCD4b* at the T_0_ generation were selected and further developed for analyses of target gene suppression and carotenoid content.

### RNAi-mediated suppression of the *OsCCD1, OsCCD4a,* and *OsCCD4b* genes, and their influences on leaf carotenoid content

**Fig. 3.**
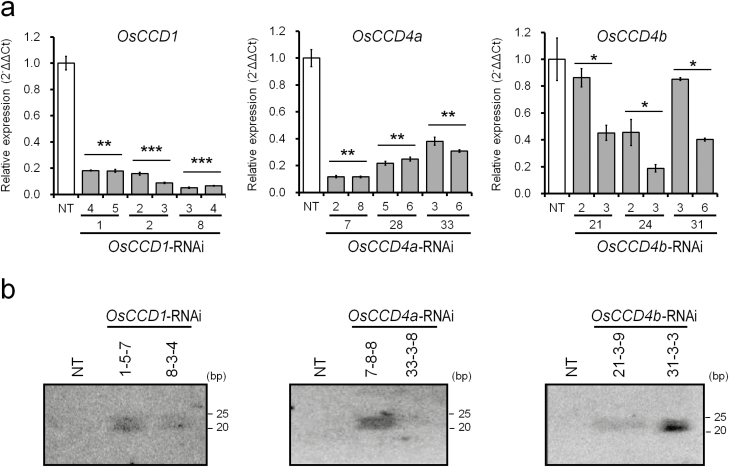
Target gene suppression in the transgenic rice lines *OsCCD1*-RNAi, *OsCCD4a*-RNAi, and *OsCCD4b*-RNAi. (a) Knock-down levels of each target gene were quantified by qRT-PCRs using T_1_ leaf tissues of two sibling lines from three independent transgenic plants for each construct. All data are the means (±SE) of triplicate measurements. The mean *C*t values were used to calculate the expression of the target gene with normalization to an internal control (*OsUbi5*) using the ΔΔ*C*t equation. The relative differences to non-transgenic (NT) plants (*Oryza sativa* cv. Ilmi) were determined using a one-tailed Student’s *t*-test: ***P<0.001, **P<0.01, *P<0.05. (b) The siRNA detection of each target gene by small RNA gel blot analysis was performed with T_2_ leaf tissues of two independent transgenic plants for each construct.

To determine whether the expression of *OsCCD1*, *OsCCD4a*, and *OsCCD4b* was suppressed by RNAi-mediated mechanisms, qRT-PCR was performed using mature leaves in two siblings of three independent T_1_ lines for *OsCCD1*-RNAi, *OsCCD4a*-RNAi, and *OsCCD4b*-RNAi (Fig. 3a). All the transgenic lines showed reduced transcript levels of *OsCCD1* (5.1–18.1%), *OsCCD4a* (8.2–23.2%), and *OsCCD4b* (18.81–86.81%) relative to non-transgenic (NT) plants. To further confirm the target gene suppression, RNA gel blot analysis was performed with mature leaves of two independent T_2_ plants for *OsCCD1*-RNAi, *OsCCD4a*-RNAi, and *OsCCD4b*-RNAi (Fig. 3b). siRNAs corresponding to the *OsCCD1*, *OsCCD4a*, and *OsCCD4b* were detected in all the transgenic plants, suggesting that RNAi-mediated suppression was well maintained from one generation to the next in the RNAi plants.

**Fig. 4.**
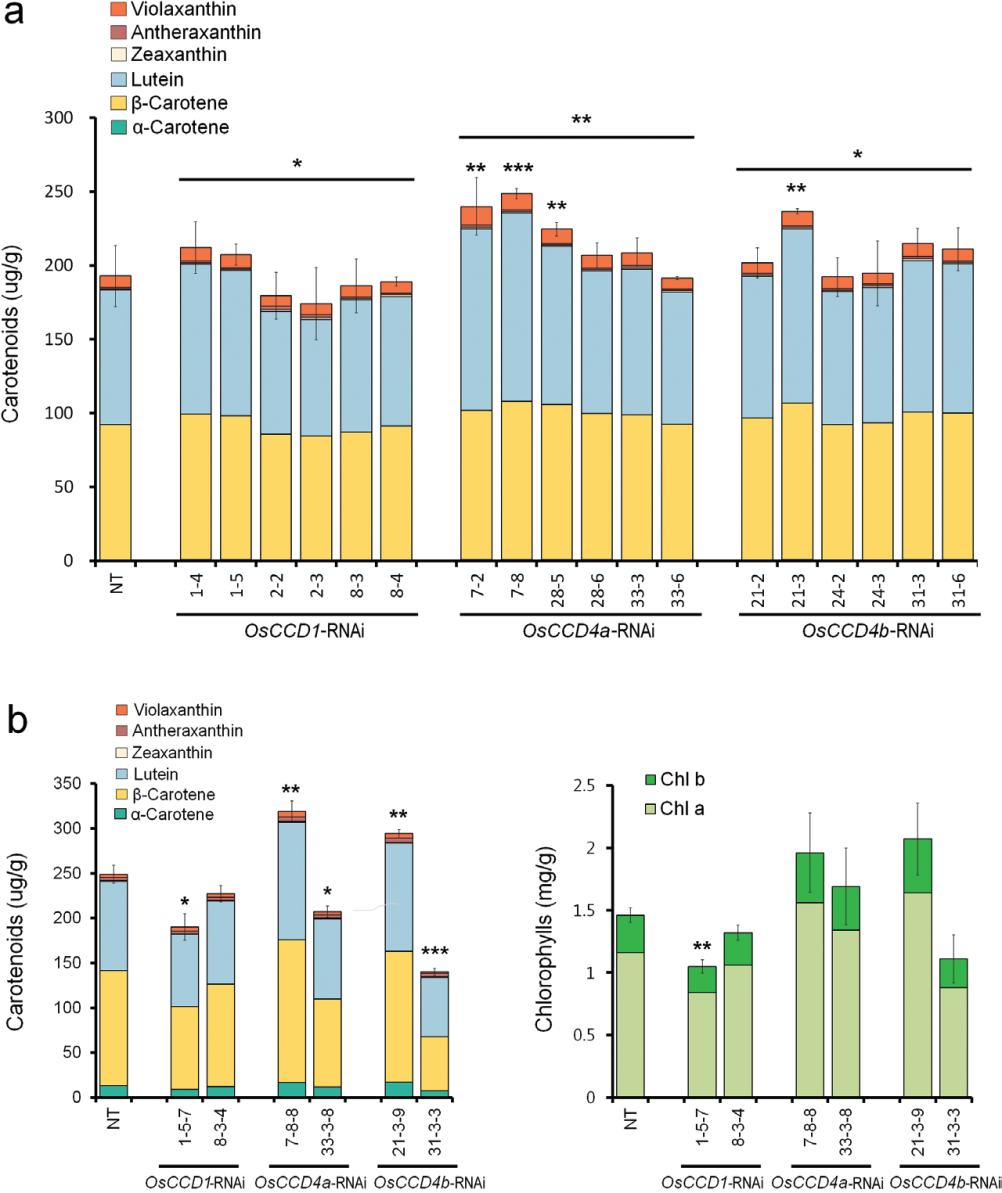
Contents and composition of leaf carotenoids in the transgenic rice lines *OsCCD1*-RNAi, *OsCCD4a*-RNAi, and *OsCCD4b*-RNAi. (a) Carotenoid levels by HPLC analysis in T_1_ leaf tissues of two sibling lines from three independent transgenic plants for each construct. (b) Levels of carotenoids, measured by HPLC, and chlorophylls, measured spectrophotometrically using absorbance, in T_2_ leaf tissues of two independent transgenic plants for each construct. NT, non-transgenic rice (*Oryza sativa* cv. Ilmi). Data are means (±SD) of three replicates. Differences relative to NT plants were determined using a one-tailed Student’s *t*-test: ***P<0.001, **P<0.01, *P<0.05.

To assess the influence of *OsCCD1*, *OsCCD4a*, and *OsCCD4b* gene suppression on leaf carotenoids, the levels of carotenoids and chlorophylls were analysed by HPLC using the same mature leaf tissue as in the RNA analyses (Fig. 4). The total amount of carotenoids in the T_1_ generation was significantly increased overall in the *OsCCD4a* lines and in the individual lines *OsCCD4a*-RNAi-*7* and -*28* and *OsCCD4b*-RNAi-*21* (Fig. 4a). Similar results were observed in the *OsCCD4a*-RNAi-*7* and *OsCCD4b*-RNAi-*21* lines in the T_2_ generation where, as well as total carotenoids, significant enhancements were found in five (α-carotene, β-carotene, lutein, antheraxanthin, and violaxanthin) and three (α-carotene, lutein, and antheraxanthin) components, respectively (Fig. 4b, Supplementary Fig. S3). The other four lines (*OsCCD1*-RNAi-*1* and -*8*, *OsCCD4a*-RNAi-*33*, and *OsCCD4b*-RNAi-*31*) had decreased carotenoid levels in the T_2_ generation relative to NT plants (Fig. 4b). Levels of chlorophylls displayed similar patterns to those of carotenoids. Suppression of either *OsCCD4a* or *OsCCD4b* had the potential to affect the accumulation of leaf carotenoids, with the effect being dependent on the transgenic line.

### Influence of *OsCCD1, OsCCD4a,* and *OsCCD4b* suppression on seed carotenoid content

To determine the effects of suppressing *OsCCD1*, *4a*, and *4b* on seed carotenoids, a strain of carotenoid-intensifying golden rice previously developed by Jeong *et al.* (2017) using a bicistronic recombinant gene *stPAC* (*stPsy:2A:Tp:stCrtI*), was used as a male parent for conventional breeding (Fig. 2b). With the homozygous *stPAC* rice having a single intact copy of the transgene, two independent lines each of *OsCCD1*-RNAi, *OsCCD4a*-RNAi, and *OsCCD4b*-RNAi were cross-fertilized as female parents after verification of their homozygosity in the T_3_ plant generation by TaqMan-PCR (Supplementary Fig. S4a). After further propagation of the resultant F_1_ seeds to the F_3_ generation, homozygosity for two transgenes between *stPAC* and each of *OsCCD1*-RNAi, *OsCCD4a*-RNAi, or *OsCCD4b*-RNAi was verified using TaqMan-PCR (Supplementary Fig. S4b).

**Fig. 5.**
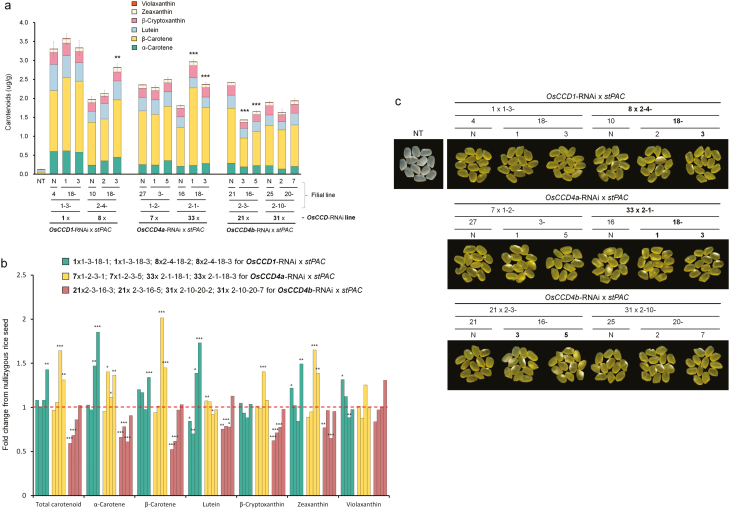
Carotenoid levels and polished color of interbred seeds of rice from two independent lines of *OsCCD1*-RNAi, *OsCCD4a*-RNAi, and *OsCCD4b*-RNAi and a *stPAC* plant displaying carotenoid-accumulating golden colored seeds. (a) Carotenoid levels, measured by HPLC, of homozygous F_4_ seeds containing two transgenes: *stPAC* and one of the RNAi genes, compared with non-transgenic (NT) plants (*Oryza sativa* cv. Ilmi). Data are means (±SD) of three replicates. The relative difference compared with each nullizygous (N) rice seed was determined using a one-tailed Student’s *t*-test. The nullizygous rice line has only a *stPAC* gene and is without the RNAi gene for each of the *OsCCD*s after being segregated from interbreeding lines (b) Fold-changes in individual carotenoid components of homozygous F_4_ seeds relative to that of each N seed. Differences between groups relative to a value of 1 were also determined using a one-tailed Student’s *t*-test. All significant differences in (a, b) are indicated as ***P<0.001, * *P*<0.01, *P<0.05. (c) Phenotypes of endosperm colors in homozygous F_4_ seeds after polishing. Line numbers with significantly enhanced carotenoid contents in (a, b) and a slightly more intense golden color compared with each N line are highlighted in bold.

To evaluate more accurately the effects on carotenoid content in rice endosperms of knock-down of the *OsCCD1*, *OsCCD4a*, and *OsCCD4b* genes, control *stPAC* seeds without T-DNA for the suppression of *OsCCD1*, *OsCCD4a*, and *OsCCD4b* were segregated out as nullizygotes from each interbreeding line. This was confirmed by the detection of two different T-DNA fragments for *stPAC* and either *OsCCD1*-RNAi, *OsCCD4a*-RNAi, or *OsCCD4b*-RNAi, by genomic PCR (Supplementary Fig. S4c). HPLC analysis of filial lines from two independent lines of *OsCCD1*-RNAi and *OsCCD4a*-RNAi showed an increase in total carotenoids overall relative to nullizygous seeds (Fig. 5a, Supplementary Table S3). Of these, representative lines that were bred from *OsCCD1*-RNAi-*8* and *OsCCD4a*-RNAi-*33* respectively displayed significant enhancements in total carotenoids of up to 1.4- and 1.6-fold compared with each nullizygous lines, with a slightly more intense golden color (Fig. 5b, c). In contrast, the intercrossed lines from *OsCCD4b*-RNAi generally had decreased levels of total carotenoids and individual components, including α-carotene, β-carotene, lutein, zeaxanthin, and β-cryptoxanthin (Fig. 5b, Supplementary Table S3). Interestingly, *OsCCD1*-RNAi and *OsCCD4a*-RNAi showed differential patterns in the enhancement of α-ring carotenoids (maximum 1.9- and 1.7-fold increases in α-carotene and lutein, respectively) and β-ring carotenoids (maximum 2.0- and 1.7-fold increases in β-carotene and zeaxanthin, respectively) (Fig. 5b). This suggested that either *OsCCD1* or *OsCCD4a* could significantly increase the levels of seed carotenoids when their expression was knocked-down, but this effect was not seen with *OsCCD4b*.

### Carotenoid-cleavage activities of OsCCD1, OsCCD4a, and OsCCD4b in carotenoid-accumulating *E. coli* strains

**Fig. 6.**
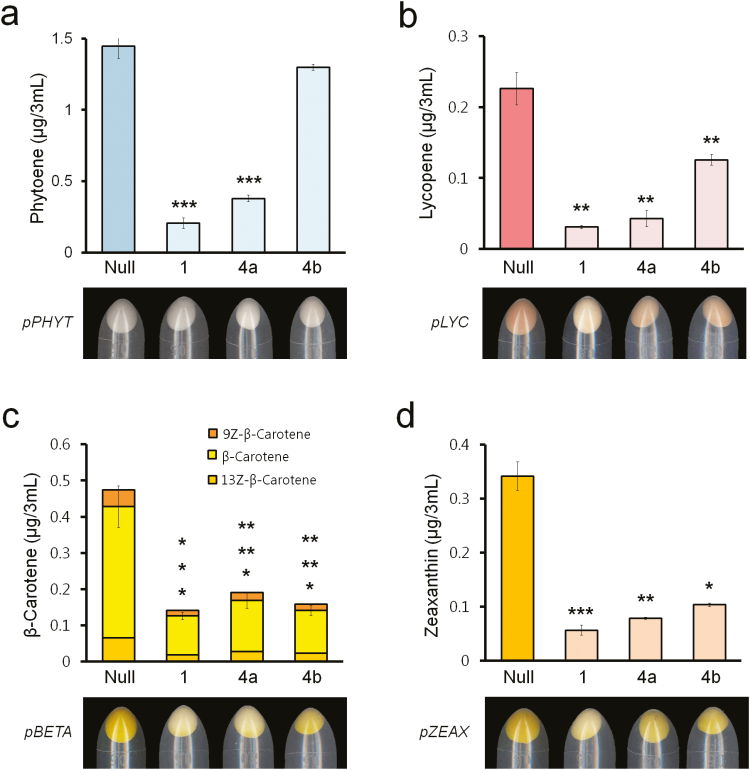
Changes in carotenoid levels and colony color according to the individual overexpression of *OsCCD1 OsCCD4a*, and *OsCCD4b* in four carotenoid-accumulating *E. coli* strains. (a) Phytoene contents measured by HPLC in a *pPHYT*-harboring *E. coli* strain. (b) Lycopene contents measured by HPLC in a *pLYC*-harboring *E. coli* strain. (c) β-Carotene contents measured by HPLC in a *pBETA*-harboring *E. coli* strain. (d) Zeaxanthin contents measured by HPLC in a *pZEAX*-harboring *E. coli* strain. Null represents each of the four vector-harboring *E. coli* strains without the rice *CCD* genes. The relative difference of each null *E. coli* strain was determined using a one-tailed Student’s *t*-test: ***P<0.001, **P<0.01, *P<0.05.

To determine whether *OsCCD1*, *OsCCD4a*, and *OsCCD4b* are involved in carotenoid degradation, three arabinose-inducible *E. coli* expression vectors were constructed with full-length cDNAs as *pOsCCD1*, *pOsCCD4a*, and *pOsCCD4b* (Supplementary Fig. S5a). They were used as competent cells for the individual transformation of four different vectors accumulating phytoene (*pPHYT*), lycopene (*pLYC*), β-carotene (*pBETA*), and zeaxanthin (*pZEAX*) (Supplementary Fig. S5b). The successful introduction of these four different carotenoid-accumulating plasmids into the *E. coli* strains harboring *pOsCCD1*, *pOsCCD4a*, and *pOsCCD4b* was confirmed by the detection of corresponding PCR fragments of *OsCCD* genes and carotenoid biosynthetic operon genes including *CrtE*, *CrtB*, *CrtI*, *CrtY*, and *CrtZ* (Supplementary Fig. S5c). The resultant *E. coli* strains were treated with arabinose to induce the transcription of *OsCCD1*, *OsCCD4a*, and *OsCCD4b* under the carotenoid-accumulating plasmids, which could display different colors depending on the production of phytoene (white), lycopene (pink), β-carotene (yellow), and zeaxanthin (yellow) as a null control (Fig. 6). Expression of the genes resulted in the display of paler colors in *E. coli* cells producing lycopene, β-carotene, and zeaxanthin except for colorless phytoene. The results showed more distinct differences in cell colors for *OsCCD1* than the other genes. Further HPLC analysis showed that the overall contents of carotenoids, except for phytoene in the case of the *OsCCD4b* gene, were significantly decreased by the expression of *OsCCD1*, *OsCCD4a*, and *OsCCD4b* relative to the null control. Among the three rice CCDs, the cleavage activity resulting from the expression of *OsCCD1* was highest in all four of the carotenoid substrates, with considerably reduced levels of phytoene (down to as low as 14%), lycopene (14%), β-carotene (30%), and zeaxanthin (16%), without substrate specificity. This was followed by *OsCCD4a* and *OsCCD4b*, except in the case of β-carotene, which showed a slightly lower cleavage efficiency in *OsCCD4a* than in *OsCCD4b*.

## Discussion

Suppression of carotenoid catabolic metabolism by CCDs has been a notable strategy to increase carotenoid content in plants by blocking their degradation into small molecules called apocarotenoids or CCPs (Gonzalez-Jorge *et al.*, 2013; Zhai *et al.*, 2016). Individual CCDs across the different types that are found in plants specifically cleave targeted carotenoid substrates at different double-bond positions to yield diverse apocarotenoids, which function as pigments, volatiles, defense compounds, and signals (Walter and Strack, 2011; Hou *et al.*, 2016). In order to increase nutritional value in crops by silencing carotenoid cleavage whilst having a minimal physiological effect on apocarotenoids, CCDs that are known to be associated with the biosynthesis of signal molecules (NCEDs for ABAs, and CCD7 and CCD8 for strigolactones) have been excluded as target candidates for genetic engineering. Thus, among the nine rice genes that are known to be orthologues of Arabidopsis and maize CCD gene families (three NCEDs: *OsNCED2*, *OsNCED3*, and *OsNCED9*; and six CCDs: *OsCCD1*, *OsCCD4a*, *OsCCD4b*, *OsCCD7*, *OsCCD8a*, and *OsCCD8b*; Vallabhaneni *et al*., 2010; Walter *et al.*, 2010), we selected *OsCCD1, OsCCD4a*, and *OsCCD4b* as potential candidates for the improvement of carotenoid content in rice.

As target genes for knock-down in rice plants, transcript abundance for *OsCCD1*, *OsCCD4a*, and *OsCCD4b* was first compared in various tissues including leaves and seeds at different development stages (Fig. 1). The results indicated that *OsCCD1* and *OsCCD4a* were preferentially expressed in the leaves, with the former having the highest level of expression, and the highest level of expression during seed development was at the early stages (10 DAF) for *OsCCD1* and during the middle stages (20 DAF) for *OsCCD4a*, suggesting that these genes needed to be silenced in leaves and seeds. The expression of *OsCCD4b* was very low in leaves and showed similar levels throughout seed development.

Molecular characteristics based on protein sequence similarities effectively distinguished between OsCCD1 and the two OsCCD4s. The latter displayed a closer relationship within a monocotyledonous CCD1 subgroup including ZmCCD1 and CsCCD1, and a monocotyledonous CCD4 subgroup including CsCCD4a, CsCCD4b, CsCCD4c, and CsZCD (known as a truncated CsCCD4 form without cleavage activity; Rubio *et al.*, 2008), than with dicotyledonous CCD1 and CCD4 subgroups (Supplementary Fig. S1a). The protein structures of OsCCD1, OsCCD4a, and OsCCD4b were analysed on the basis of comparative modeling results of crocus CsCCD4c via structural alignment with maize VP14 as the NCED form (Messing *et al.*, 2010; Rubio-Moraga *et al.*, 2014). All three rice CCDs had key residues recognized for VP14 activity from crystal structure studies, including two Phe residues of a broad CCD group, either Phe or Leu as a distinguishable residue between a narrow CCD and NCED group, and Trp and Leu, Ser, and Pro, and Ala and Pro residues between the CCD1 and CCD4 subgroups (Supplementary Fig. S1b). In addition, Supplementary Fig. S1b and Fig. 2a demonstrate good conservation in the position and numbers of four histidine residues that act as typical ligands of a non-heme iron co-factor that is required for oxygenase activity and three glutamate residues that fix the iron-ligating histidine by hydrogen bonds, supporting their enzymatic activities as either a CCD1 or CCD4 subgroup (Schwartz *et al.*, 1997; Kloer and Schulz, 2006).

In previous studies, up-regulation of either *CCD1* or *CCD4* has been shown to lead to less pigmentation in flowers of *Chrysanthemum* (*CmCCD4a*), *Oncidium* (*OgCCD1*), and *Lilium* (*LbCCD4*), in fruits of peach (*PpCCD4*), in tubers of potato (*StCCD4*), in the endosperm of maize (*ZmCCD1*), and in the seed of Arabidopsis (*AtCCD4*). The reduced levels of carotenoids suggest that they are used as substrates for CCD activity (Ohmiya *et al.*, 2006; Campbell *et al.*, 2010; Chiou *et al.*, 2010; Vallabhaneni *et al.*, 2010; Brandi *et al.*, 2011; Hai *et al.*, 2012; Gonzalez-Jorge *et al.*, 2013; Bai *et al.*, 2016). They also influence flavors, pigments, or signals in flower petals of petunia (*PhCCD1*) and rose (*RdCCD1* and *RdCCD4*), in flower stigmas of crocus (*CsCCD1b*, *CsCCD2*, *CsCCD4a*, *CsCCD4b*, and *CsCCD4c*), in fruits of tomato (*LeCCD1*), strawberry (*FaCCD1*), and citrus (*CitCCD4* and *CitCCD4b1*), and in tubers of potato (*StCCD4*), implying that CCD action generates a diverse number of apocarotenoids (Simkin *et al.*, 2004*a*, 2004*b*; García-Limones *et al.* 2008; Rubio *et al.* 2008; Huang *et al.*, 2009*a*, 2009*b*; Ma *et al.*, 2013; Rodrigo *et al.*, 2013; Frusciante *et al.*, 2014; Rubio-Moraga *et al.*, 2014; Bruno *et al.*, 2015).

To assess the ability of *OsCCD1*, *OsCCD4a*, and *OsCCD4b* in enhancing the nutritional value of crop plants by increasing carotenoid content, RNAi-mediated suppression was performed in transgenic rice plants (Supplementary Fig. S2, Fig. 3). Among these, the sequential generation of T_1_ and T_2_ of the *OsCCD4a*-RNAi-*7* lines showed a reliable increase in the leaf carotenoids α-carotene, β-carotene, lutein, antheraxanthin, and violaxanthin (but not zeaxanthin) relative to non-transgenic (NT) plants (Fig. 4, Supplementary Fig. S3). The effects of the *OsCCD1*-RNAi, *OsCCD4a*-RNAi, and *OsCCD4b*-RNAi lines on seed carotenoids were ascertained via conventional breeding with a rice strain that had previously been developed using a modified recombinant *stPAC* (*stPsy:2A:Tp:stCrtI*) gene via codon-optimization for rice on the basis of a bicistronic recombinant *PAC* (*Psy:2A:Tp:CrtI*) gene with the ability to produce carotenoids in the endosperm (Ha *et al.*, 2010; Jeong *et al.*, 2017). From homozygous F_3_ plants that were self-fertilized from F_1_ hybrid seeds between *OsCCDs*-RNAi lines and a *stPAC 25* line (Supplementary Fig. S4), F_4_ seeds descended from the line *OsCCD1*-RNAi-*8* displayed a 1.4-fold increase in total carotenoids, with an α-ring carotenoid preference in the *2-4-18-3* line relative to the *2-4-10-N* (nullizygous) line. Filial seeds from *OsCCD4a*-RNAi-*33* showed an even higher increase of 1.6-fold in total carotenoids in the *2-1-18-1* line relative to the *2-1-16-N* line, with a β-ring carotenoid preference, including β-carotene, β-cryptoxanthin, and zeaxanthin (Fig. 5, Supplementary Table S3), suggesting that they had greater nutritional value as crop plants.

To assess the carotenoid cleaving activity of OsCCD1, OsCCD4a, and OsCCD4b, their genes were overexpressed in four *E. coli* systems supplying different substrates of phytoene, lycopene, β-carotene, and zeaxanthin by harboring *pPHYT*, *pLYC*, *pBETA*, and *pZEAX*, respectively (Supplementary Fig. S5). The results indicated that the expression of *OsCCD1*, *OsCCD4a*, and *OsCCD4b* lowered the carotenoid levels and lessened the color of the *E. coli* pellet, suggesting that carotenoids were used as substrates, and with similar relatively high activities of *OsCCD1* and *OsCCD4a* compared to *OsCCD4b* without substrate specificity (Fig. 6). Our results and those of a previous study using several carotenoids and apocarotenoids as substrates suggest that OsCCD1 has cleaving activity at diverse double-bond sites, namely C5–C6 ⁄ C5′–C6′, C7–C8 ⁄ C7′–C8′ and C9–C10 ⁄ C9′–C10′ (Ilg *et al.*, 2009). OsCCD1 might have strong carotenoid cleaving activity, and OsCCD4a could also be expected to have an equivalent capability (Fig. 6).

In studies using transgenic approaches, the overexpression of either *CCD1* or *CCD4* to produce apocarotenoid compounds functioning as signaling and volatile molecules has been reported in only a limited number of cases, for example *LeCCD1A* in petunia, *OsCCD1* in Golden rice, *VvCCD1* in grapevine, and *AtCCD4* in rice plants, even though this could control plant physiology including development, biotic stress resistance, and abiotic stress tolerance, and enhance floral scents and fruit flavors (Simkin *et al.*, 2004b;Ilg *et al.*, 2010; Lashbrooke *et al.*, 2013; Song *et al.*, 2016). Only the heterologous *AtCCD4* gene in rice leaves has resulted fewer carotenoids, with a reduction of 26% and a 2-fold increase of β-ionone. Both and have been noted as targets that could be blocked to improve the nutritional value in major food crops and the color intensity in fruit and flowering crops, for example via co-suppression of *PhCCD1* through overexpression of *LeCCD1A* in petunia, antisense expression of *LeCCD1* in tomato and *OsCCD1* in Golden rice, RNAi of *StCCD4* in potato and *VvCCD1* in grapevine, virus-induced silencing of *PpCCD4* in peach, gamma-irradiation of *CmCCD4a* in chrysanthemum, and CRISPR/Cas9-mediated mutagenesis of either *OsCCD4a* or *OsCCD4b* in rice and *InCCD4* in Japanese morning glory (Simkin *et al.*, 2004*a*, 2004*b*; Campbell *et al.*, 2010; Ilg *et al.*, 2010; Lashbrooke *et al.*, 2013; Bai *et al.*, 2016; Jo *et al.*, 2016; Yang *et al.*, 2017; Watanabe *et al.*, 2018). Among these suppression studies, *StCCD4* in potato tubers, *PpCCD4* in peach fruits, and *CmCCD4a* and *InCCD4* in chrysanthemum and in Japanese morning glory petals resulted in an enhanced yellow coloration due to the increase of carotenoid content, and *PhCCD1* in petunia caused a maximum 76% decrease in β-ionone synthesis in flower corollas (Simkin et al., 2004*b*; Campbell et al., 2010; Bai *et al.*, 2016; Jo *et al.*, 2016; Watanabe *et al.*, 2018). In contrast, suppression of *LeCCD1* in tomato, *OsCCD1* in Golden rice, and *OsCCD4a* and *OsCCD4b* in rice showed no correlation with enhanced carotenoid accumulation (Simkin *et al.*, 2004*a*; Ilg *et al.*, 2010; Yang *et al.*, 2017). In our current study, even antisense expression of *OsCCD1* seemed to slightly decrease the total content of carotenoids in a Golden rice background, but suppressed levels of *OsCCD1* transcripts still need to be observed. In addition, *OsCCD4a* and *OsCCD4b* remain to be knocked-out in carotenoid-accumulating rice rather than in common rice, which is ineffectual for carotenoid biosynthesis.

The potential to enhance nutritionally valuable carotenoids by preventing their transition into apocarotenoids has been consistently proposed for major food crops including maize, rice, and wheat (Ilg *et al.*, 2010; Gonzalez-Jorge *et al.*, 2013; da Silva *et al*., 2014; Qin *et al.*, 2016; Yang *et al.*, 2017). Silencing of CCD genes *in planta* has already successfully resulted in affecting color traits in potato tubers, peach fruits, and chrysanthemum and Japanese morning glory flowers (Campbell *et al.*, 2010; Bai *et al.*, 2016; Jo *et al.*, 2016; Watanabe *et al.*, 2018).In this study, we used a knock-down strategy to demonstrate that *OsCCD4a* could enhance the contents of valuable provitamin A components in carotenoid-abundant rice leaves and carotenoid-accumulating rice seeds in the form of β-carotenoids. For practical use, a more powerful tool in the knock-out strategy is needed to completely turn off the function of *OsCCD4a*. In addition, the simultaneous silencing of *OsCCD4a* and *OsCCD1* might be effective in further increasing the content of carotenoids in rice endosperm when accompanied with genetic engineering for carotenoid biosynthesis, as the potential of *OsCCD1* as another promising target for crop biofortification remains considerable.

## Supplementary data

Supplementary data are available at *JXB* online.

Table S1. Primer sequence information for experiments *in planta* including quantitative real-time PCR, binary vector construction, and genomic DNA PCR.

Table S2. Primer sequence information for experiments *in vitro* including expression vector construction and colony PCR.

Table S3. Carotenoid content and composition of interbred filial seeds as measured by HPLC.

Fig. S1. Phylogenetic tree and alignment among plant CCDs on the basis of amino acid sequence similarities.

Fig. S2. Verification of transgenic rice lines of *OsCCD1*-RNAi, *OsCCD4a*-RNAi, and *OsCCD4b*-RNAi.

Fig. S3. Contents of individual carotenoid components in transgenic rice leaf tissues of *OsCCD1*-RNAi, *OsCCD4a*-RNAi, and *OsCCD4b*-RNAi.

Fig. S4. Verification of transgene homozygosity in interbred rice lines between each of two independent lines for *OsCCD1*-RNAi, *OsCCD4a*-RNAi, and *OsCCD4b*-RNAi (female parent) and a *stPAC* line (male parent).

Fig. S5. *In vitro* expression of *OsCCD1, OsCCD4a*, and *OsCCD4b* in four carotenoid-accumulating *E. coli* strains to analyse carotenoid-cleavage activities.

Supplementary Tables and Figures

## References

[CIT0001] Auldridge ME, McCarty DR, Klee HJ 2006 Plant carotenoid cleavage oxygenases and their apocarotenoid products. Current Opinion in Plant Biology 9, 315–321. 16616608 10.1016/j.pbi.2006.03.005

[CIT0002] Bai S, Tuan PA, Tatsuki M, Yaegaki H, Ohmiya A, Yamamizo C, Moriguchi T 2016 Knockdown of carotenoid cleavage dioxygenase 4 (CCD4) via virus-induced gene silencing confers yellow coloration in peach fruit: evaluation of gene function related to fruit traits. Plant Molecular Biology Reporter 34, 257–264.

[CIT0003] Brandi F, Bar E, Mourgues F, Horváth G, Turcsi E, Giuliano G, Liverani A, Tartarini S, Lewinsohn E, Rosati C 2011 Study of ‘Redhaven’ peach and its white-fleshed mutant suggests a key role of CCD4 carotenoid dioxygenase in carotenoid and norisoprenoid volatile metabolism. BMC Plant Biology 11, 24. 21269483 10.1186/1471-2229-11-24 PMC3045293

[CIT0004] Bruno M, Beyer P, Al-Babili S 2015 The potato carotenoid cleavage dioxygenase 4 catalyzes a single cleavage of β-ionone ring-containing carotenes and non-epoxidated xanthophylls. Archives of Biochemistry and Biophysics 572, 126–133. 25703194 10.1016/j.abb.2015.02.011

[CIT0005] Campbell R, Ducreux LJ, Morris WL, Morris JA, Suttle JC, Ramsay G, Bryan GJ, Hedley PE, Taylor MA 2010 The metabolic and developmental roles of carotenoid cleavage dioxygenase4 from potato. Plant Physiology 154, 656–664. 20688977 10.1104/pp.110.158733 PMC2949026

[CIT0006] Chiou CY, Pan HA, Chuang YN, Yeh KW 2010 Differential expression of carotenoid-related genes determines diversified carotenoid coloration in floral tissues of *Oncidium cultivars*. Planta 232, 937–948. 20635095 10.1007/s00425-010-1222-x

[CIT0007] Cunningham FX Jr, Pogson B, Sun Z, McDonald KA, DellaPenna D, Gantt E 1996 Functional analysis of the beta and epsilon lycopene cyclase enzymes of *Arabidopsis* reveals a mechanism for control of cyclic carotenoid formation. The Plant Cell 8, 1613–1626. 8837512 10.1105/tpc.8.9.1613 PMC161302

[CIT0008] da Silva Messias R, Galli V, Dos Anjos E Silva SD, Rombaldi CV 2014 Carotenoid biosynthetic and catabolic pathways: gene expression and carotenoid content in grains of maize landraces. Nutrients 6, 546–563. 24476639 10.3390/nu6020546 PMC3942716

[CIT0009] Diretto G, Tavazza R, Welsch R, Pizzichini D, Mourgues F, Papacchioli V, Beyer P, Giuliano G 2006 Metabolic engineering of potato tuber carotenoids through tuber-specific silencing of lycopene epsilon cyclase. BMC Plant Biology 6, 13. 16800876 10.1186/1471-2229-6-13 PMC1570464

[CIT0010] Frusciante S, Diretto G, Bruno M, et al 2014 Novel carotenoid cleavage dioxygenase catalyzes the first dedicated step in saffron crocin biosynthesis. Proceeding of the National Academy of Sciences, USA 111, 12246–12251. 10.1073/pnas.1404629111 PMC4143034 25097262

[CIT0011] García-Limones C, Schnäbele K, Blanco-Portales R, Luz Bellido M, Caballero JL, Schwab W, Muñoz-Blanco J 2008 Functional characterization of FaCCD1: a carotenoid cleavage dioxygenase from strawberry involved in lutein degradation during fruit ripening. Journal of Agricultural and Food Chemistry 56, 9277–9285. 18778069 10.1021/jf801096t

[CIT0012] Giuliano G 2017 Provitamin A biofortification of crop plants: a gold rush with many miners. Current Opinion in Biotechnology 44, 169–180. 28254681 10.1016/j.copbio.2017.02.001

[CIT0013] Giuliano G, Al-Babili S, von Lintig J 2003 Carotenoid oxygenases: cleave it or leave it. Trends in Plant Science 8, 145–149. 12711223 10.1016/S1360-1385(03)00053-0

[CIT0014] Gonzalez-Jorge S, Ha SH, Magallanes-Lundback M, et al 2013 Carotenoid cleavage dioxygenase4 is a negative regulator of β-carotene content in *Arabidopsis* seeds. The Plant Cell 25, 4812–4826. 24368792 10.1105/tpc.113.119677 PMC3903989

[CIT0015] Ha SH, Liang YS, Jung H, Ahn MJ, Suh SC, Kweon SJ, Kim DH, Kim YM, Kim JK 2010 Application of two bicistronic systems involving 2A and IRES sequences to the biosynthesis of carotenoids in rice endosperm. Plant Biotechnology Journal 8, 928–938. 20649940 10.1111/j.1467-7652.2010.00543.x

[CIT0016] Hai NTL, Masuda J-I, Miyajima I, Thien NQ, Mojtahedi N, Hiramatsu M, Kim J-H, Okubo H 2012 Involvement of carotenoid cleavage dioxygenase 4 gene in tepal color change in *Lilium brownii* var. *colchesteri*. Journal of the Japanese Society for Horticultural Science 81, 366–373.

[CIT0017] Hou X, Rivers J, León P, McQuinn RP, Pogson BJ 2016 Synthesis and function of apocarotenoid signals in plants. Trends in Plant Science 21, 792–803. 27344539 10.1016/j.tplants.2016.06.001

[CIT0018] Huang FC, Horváth G, Molnár P, Turcsi E, Deli J, Schrader J, Sandmann G, Schmidt H, Schwab W 2009a Substrate promiscuity of RdCCD1, a carotenoid cleavage oxygenase from *Rosa damascena*. Phytochemistry 70, 457–464. 19264332 10.1016/j.phytochem.2009.01.020

[CIT0019] Huang FC, Molnár P, Schwab W 2009b Cloning and functional characterization of carotenoid cleavage dioxygenase 4 genes. Journal of Experimental Botany 60, 3011–3022. 19436048 10.1093/jxb/erp137 PMC2718213

[CIT0020] Ilg A, Beyer P, Al-Babili S 2009 Characterization of the rice carotenoid cleavage dioxygenase 1 reveals a novel route for geranial biosynthesis. The FEBS Journal 276, 736–747. 19120446 10.1111/j.1742-4658.2008.06820.x

[CIT0021] Ilg A, Yu Q, Schaub P, Beyer P, Al-Babili S 2010 Overexpression of the rice *carotenoid cleavage dioxygenase 1* gene in Golden Rice endosperm suggests apocarotenoids as substrates *in planta*. Planta 232, 691–699. 20549230 10.1007/s00425-010-1205-y

[CIT0022] Jeong YS, Ku H-K, Kim JK, You MK, Lim S-H, Kim J-K, Ha S-H 2017 Effect of codon optimization on the enhancement of the β-carotene contents in rice endosperm. Plant Biotechnology Reports 11, 171–179.

[CIT0023] Jo YD, Kim Y-S, Ryu J, Choi HI, Kin SW, Kang HS, Ahn J-W, Kim J-B, Kang S-Y, Kim SH 2016 Deletion of *carotenoid cleavage dioxygenase 4a* (*CmCCD4a*) and global up-regulation of plastid protein-coding genes in a mutant chrysanthemum cultivar producing yellow petals. Scientia Horticulturae 212, 49–59.

[CIT0024] Kloer DP, Schulz GE 2006 Structural and biological aspects of carotenoid cleavage. Cellular and Molecular Life Sciences 63, 2291–2303. 16909205 10.1007/s00018-006-6176-6 PMC11136134

[CIT0025] Lashbrooke JG, Young PR, Dockrall SJ, Vasanth K, Vivier MA 2013 Functional characterisation of three members of the *Vitis vinifera* L. carotenoid cleavage dioxygenase gene family. BMC Plant Biology 13, 156. 24106789 10.1186/1471-2229-13-156 PMC3854447

[CIT0026] Ma G, Zhang L, Matsuta A, Matsutani K, Yamawaki K, Yahata M, Wahyudi A, Motohashi R, Kato M 2013 Enzymatic formation of β-citraurin from β-cryptoxanthin and zeaxanthin by carotenoid cleavage dioxygenase4 in the flavedo of citrus fruit. Plant Physiology 163, 682–695. 23966550 10.1104/pp.113.223297 PMC3793050

[CIT0027] Martin RC, Liu P-P, Nonogaki H 2005 Simple purification of small RNAs from seeds and efficient detection of multiple microRNAs expressed in *Arabidopsis thaliana* and tomato (*Lycopersicon esculentum*) seeds. Seed Science Reserch 15, 319–328.

[CIT0028] Mayer JE, Pfeiffer WH, Beyer P 2008 Biofortified crops to alleviate micronutrient malnutrition. Current Opinion in Plant Biology 11, 166–170. 18314378 10.1016/j.pbi.2008.01.007

[CIT0029] Messing SA, Gabelli SB, Echeverria I, Vogel JT, Guan JC, Tan BC, Klee HJ, McCarty DR, Amzel LM 2010 Structural insights into maize viviparous14, a key enzyme in the biosynthesis of the phytohormone abscisic acid. The Plant Cell 22, 2970–2980. 20884803 10.1105/tpc.110.074815 PMC2965545

[CIT0030] Miki D, Shimamoto K 2004 Simple RNAi vectors for stable and transient suppression of gene function in rice. Plant & Cell Physiology 45, 490–495. 15111724 10.1093/pcp/pch048

[CIT0031] Nisar N, Li L, Lu S, Khin NC, Pogson BJ 2015 Carotenoid metabolism in plants. Molecular Plant 8, 68–82. 25578273 10.1016/j.molp.2014.12.007

[CIT0032] Ohmiya A, Kishimoto S, Aida R, Yoshioka S, Sumitomo K 2006 Carotenoid cleavage dioxygenase (CmCCD4a) contributes to white color formation in chrysanthemum petals. Plant Physiology 142, 1193–1201. 16980560 10.1104/pp.106.087130 PMC1630759

[CIT0033] Pons E, Alquézar B, Rodríguez A, Martorell P, Genovés S, Ramón D, Rodrigo MJ, Zacarías L, Peña L 2014 Metabolic engineering of β-carotene in orange fruit increases its *in vivo* antioxidant properties. Plant Biotechnology Journal 12, 17–27. 24034339 10.1111/pbi.12112

[CIT0034] Qin X, Fischer K, Yu S, Dubcovsky J, Tian L 2016 Distinct expression and function of carotenoid metabolic genes and homoeologs in developing wheat grains. BMC Plant Biology 16, 155. 27405473 10.1186/s12870-016-0848-7 PMC4943016

[CIT0035] Rodrigo MJ, Alquézar B, Alós E, Medina V, Carmona L, Bruno M, Al-Babili S, Zacarías L 2013 A novel carotenoid cleavage activity involved in the biosynthesis of Citrus fruit-specific apocarotenoid pigments. Journal of Experimental Botany 64, 4461–4478. 24006419 10.1093/jxb/ert260 PMC3808326

[CIT0036] Römer S, Lübeck J, Kauder F, Steiger S, Adomat C, Sandmann G 2002 Genetic engineering of a zeaxanthin-rich potato by antisense inactivation and co-suppression of carotenoid epoxidation. Metabolic Engineering 4, 263–272. 12646321 10.1006/mben.2002.0234

[CIT0037] Rubio A, Rambla JL, Santaella M, Gómez MD, Orzaez D, Granell A, Gómez-Gómez L 2008 Cytosolic and plastoglobule-targeted carotenoid dioxygenases from *Crocus sativus* are both involved in beta-ionone release. The Journal of Biological Chemistry 283, 24816–24825. 18611853 10.1074/jbc.M804000200 PMC3259819

[CIT0038] Rubio-Moraga A, Rambla JL, Fernández-de-Carmen A, Trapero-Mozos A, Ahrazem O, Orzáez D, Granell A, Gómez-Gómez L 2014 New target carotenoids for CCD4 enzymes are revealed with the characterization of a novel stress-induced carotenoid cleavage dioxygenase gene from *Crocus sativus*. Plant Molecular Biology 86, 555–569. 25204497 10.1007/s11103-014-0250-5

[CIT0039] Schwartz SH, Tan BC, Gage DA, Zeevaart JA, McCarty DR 1997 Specific oxidative cleavage of carotenoids by VP14 of maize. Science 276, 1872–1874. 9188535 10.1126/science.276.5320.1872

[CIT0040] Simkin AJ, Schwartz SH, Auldridge M, Taylor MG, Klee HJ 2004a The tomato carotenoid cleavage dioxygenase 1 genes contribute to the formation of the flavor volatiles beta-ionone, pseudoionone, and geranylacetone. The Plant Journal 40, 882–892. 15584954 10.1111/j.1365-313X.2004.02263.x

[CIT0041] Simkin AJ, Underwood BA, Auldridge M, Loucas HM, Shibuya K, Schmelz E, Clark DG, Klee HJ 2004b Circadian regulation of the PhCCD1 carotenoid cleavage dioxygenase controls emission of beta-ionone, a fragrance volatile of petunia flowers. Plant Physiology 136, 3504–3514. 15516502 10.1104/pp.104.049718 PMC527150

[CIT0042] Sommer A, Vyas KS 2012 A global clinical view on vitamin A and carotenoids. The American Journal of Clinical Nutrition 96, 1204S–1206S. 23053551 10.3945/ajcn.112.034868

[CIT0043] Song M-H, Lim S-H, Kim JK, Jung ES, John KM, You M-K, Ahn S-N, Lee CH, Ha S-H 2016 *In planta* cleavage of carotenoids by Arabidopsis carotenoid cleavage dioxygenase 4 in transgenic rice plants. Plant Biotechnology Reports 10, 291–300.

[CIT0044] Vallabhaneni R, Bradbury LM, Wurtzel ET 2010 The carotenoid dioxygenase gene family in maize, sorghum, and rice. Archives of Biochemistry and Biophysics 504, 104–111. 20670614 10.1016/j.abb.2010.07.019 PMC2957549

[CIT0045] Walter MH, Floss DS, Strack D 2010 Apocarotenoids: hormones, mycorrhizal metabolites and aroma volatiles. Planta 232, 1–17. 20396903 10.1007/s00425-010-1156-3

[CIT0046] Walter MH, Strack D 2011 Carotenoids and their cleavage products: biosynthesis and functions. Natural Product Reports 28, 663–692. 21321752 10.1039/c0np00036a

[CIT0047] Watanabe K, Oda-Yamamizo C, Sage-Ono K, Ohmiya A, Ono M 2018 Alteration of flower colour in *Ipomoea nil* through CRISPR/Cas9-mediated mutagenesis of *carotenoid cleavage dioxygenase 4*. Transgenic Research 27, 25–38. 29247330 10.1007/s11248-017-0051-0

[CIT0048] Yang X, Chen L, He J, Yu W 2017 Knocking out of carotenoid catabolic genes in rice fails to boost carotenoid accumulation, but reveals a mutation in strigolactone biosynthesis. Plant Cell Reports 36, 1533–1545. 28676963 10.1007/s00299-017-2172-6

[CIT0049] Zeng J, Wang X, Miao Y, et al 2015 Metabolic engineering of wheat provitamin A by simultaneously overexpressing *CrtB* and silencing carotenoid hydroxylase (*TaHyd*). Journal of Agricultural and Food Chemistry 63, 9083–9092. 26424551 10.1021/acs.jafc.5b04279

[CIT0050] Zhai S, Xia X, He Z 2016 Carotenoids in staple cereals: metabolism, regulation, and genetic manipulation. Frontiers in Plant Science 7, 1197. 27559339 10.3389/fpls.2016.01197 PMC4978713

