# Supplementary material for: RNAi-mediated suppression of three carotenoid-cleavage dioxygenase genes, OsCCD1, 4a, and 4b, increases carotenoid content in rice
Source: J Exp Bot. 2018 Aug 14;69(21):5105–16. doi: 10.1093/jxb/ery300 (PMC6184605; doi:10.1093/jxb/ery300)
Supplement: Supplementary Tables and Figures [file ery300_suppl_supplementary_tables-s1-s3_figures-s1-s5.pdf]

Supplementary Data

Supplementary Table S1. Primer sequence information for experiments *in planta* including quantitative real-time PCR, binary vector construction, and genomic DNA PCR.

Gene	Primer sequence (forward/reverse)	Product size (bp)
Quantitative RT-PCR		
*OsCCD1*_Ri	5'-TCTGGAGAAGAAGATGATGGTTAT-3'/<br>5'-GGGAACTCGGCTTGGTAG-3'	138
*OsCCD4a*_Ri	5'-CGGGCTTCAACATCATGC-3'/<br>5'-TAGCTCCATGTGCTCCAG-3'	116
*OsCCD4b*_Ri	5'-TTGATCTCGTCGGCTCTG-3'/<br>5'-ACCACTATCCTCGTTTCCATAC-3'	122
OsUbi5	5'-GAAGTAAGGAAGGAGGAGGA-3'/<br>5'-AAGGTGTTCAGTTCCAAGG-3'	100
Gateway vector cloning and genomic PCR		
*OsCCD1*_Ri	5'- AAAAAGCAGGCTGCAAAACAATCAGCGTG-3'/<br>5'- AGAAAGCTGGGTCCCTCCCTTAATAAGTA-3'	258
*OsCCD4a*_Ri	5'- AAAAAGCAGGCTCAGTCACAGCACCAATG-3'/<br>5'- AGAAAGCTGGGTCTCGAAGTATGTGTGCA-3'	177
*OsCCD4b*_Ri	5'- AAAAAGCAGGCTCTTCTCGCACAACAATA-3'/<br>5'- AGAAAGCTGGGTGATGTGAAATGACAACC-3'	226
attB	5'-GGGGACAAGTTTGTACAAAAAAGCAGGCT-3'/<br>5'-GGGGACCACTTTGTACAAGAAAGCTGGGT-3'	

Supplementary Table S2. Primer sequence information for experiments *in vitro* including expression vector construction and colony PCR.

Gene	Primer sequence (forward/reverse)	Product size (bp)
Gateway vector cloning and colony PCR		
OsCCD1	5’-AAAAAGCAGGCTGTATGGGAGGCGGCGATG-3’/<br>5’-AGAAAGCTGGGTGTCACGCTGATTGTTTTG-3’	1623
OsCCD4a	5’-AAAAAGCAGGCTGCATGCAAAGGATTTGCC-3’/<br>5’-AGAAAGCTGGGTATCATTGGTGCTGTGACT-3’	1917
OsCCD4b	5’-AAAAAGCAGGCTTAATGGAGGTACCCATTG-3’/<br>5’-AGAAAGCTGGGTCTTATTGTTGTGCGAGAA-3’	1731
Colony PCR		
CrtE	5'- AATGGTGAGTGGCAGTAAAGC -3‘/<br>5'- TCATGACCGGTGACCAGTCGGCAAG -3'	941
CrtB	5'-AATGAGCCAACCGCCGCTGCTTGAC-3‘/<br>5'-CTAAACGGGACGCTGCCAAAGACC -3'	931
CrtI	5'- CCGTTGTGATTGGCGCAGGCTTTG-3‘/<br>5'- TCAGGCTGGCGGTGGCTTTCG-3'	1112
CrtY	5'- GGCCTGGCCAACGGGCTGATCG-3‘/<br>5'- GCCAGGCTTCGCCCAGCGGAACC-3'	1090
CrtZ	5'- CTTGAGCGTTATTGCGATGG-3‘/<br>5'- TTATTCGGGCGAAGACGACGAGGG -3'	505

Supplementary Table S3. Carotenoid content and composition of interbred filial seeds as measured by HPLC.

			α-Carotene (%)	β-Carotene (%)	Lutein (%)	β-Cryptoxanthin (%)	Zeaxanthin (%)	Violaxanthin (%)	Total
NT	IM	Subline	0.00 (0)	0.05 ± 0.01 (42.8)	0.06 ± 0.00 (51.2)	0.00 (0)	0.005 ± 0.00 (3.9)	0.003 ± 0.00 (2.1)	0.13 ± 0.02
***OsCCD1*_Ri** x *stPAC*	**1** x 1-3-4	N	0.60 ± 0.04 (18.0)	1.60 ± 0.12 (48.5)	0.69 ± 0.02 (20.9)	0.31 ± 0.02 (9.5)	0.09 ± 0.00 (2.7)	0.01 ± 0.00 (0.3)	3.31 ± 0.21
	**1** x 1-3-18-	1	0.61 ± 0.03 (17.1)	1.93 ± 0.08 (53.9)	0.58 ± 0.02 (16.3)	0.33 ± 0.01 (9.2)	0.11 ± 0.00 (3.0)	0.01 ± 0.00 (0.4)	3.58 ± 0.14
		3	0.58 ± 0.04 (17.4)	1.87 ± 0.12 (56.1)	0.49 ± 0.02 (14.5)	0.30 ± 0.02 (8.8)	0.09 ± 0.01 (2.7)	0.01 ± 0.00 (0.4)	3.34 ± 0.20
	**8** x 2-4-10	N	0.24 ± 0.01 (12.1)	1.13 ± 0.05 (57.3)	0.29 ± 0.01 (14.7)	0.23 ± 0.01 (11.5)	0.08 ±0.00 (3.9)	0.01 ± 0.00 (0.5)	1.97 ± 0.09
	**8** x 2-4-18-	2	0.35 ± 0.01 (16.5)	1.10 ± 0.05 (51.8)	0.40 ± 0.03 (18.9)	0.20 ± 0.00 (9.4)	0.06 ± 0.01 (3.0)	0.01 ± 0.00 (0.4)	2.13 ± 0.10
		3	0.44 ± 0.02 (15.7)	1.51 ± 0.06 (53.7)	0.50 ± 0.02 (17.8)	0.24 ± 0.01 (8.4)	0.11 ±0.00 (4.0)	0.01 ± 0.00 (0.3)	2.82 ± 0.11
***OsCCD4a*_Ri** x *stPAC*	**7** x 1-2-27	N	0.25 ± 0.01 (10.8)	1.41 ± 0.01 (59.9)	0.36 ± 0.00 (15.1)	0.24 ± 0.00 (10.2)	0.08 ±0.00 (3.5)	0.01 ± 0.00 (0.5)	2.36 ± 0.01
	**7** x 1-2-3-	1	0.24 ± 0.01 (10.6)	1.33 ± 0.06 (58.2)	0.38 ± 0.00 (16.7)	0.25 ± 0.01 (10.7)	0.07 ±0.00 (3.3)	0.01 ± 0.00 (0.5)	2.29 ± 0.08
		5	0.36 ± 0.01 (14.3)	1.43 ± 0.04 (57.4)	0.38 ± 0.02 (15.2)	0.24 ± 0.01 (9.6)	0.08 ±0.00 (3.2)	0.01 ± 0.00 (0.4)	2.50 ± 0.07
	**33** x 2-1-16	N	0.21 ± 0.00 (11.4)	1.02 ± 0.02 (56.2)	0.29 ± 0.01 (16.2)	0.23 ± 0.00 (12.6)	0.06 ± 0.00 (3.2)	0.01 ± 0.00 (0.4)	1.81 ± 0.03
	**33** x 2-1-18-	1	0.23 ± 0.01 (7.7)	2.04 ± 0.05 (68.9)	0.27 ± 0.00 (9.0)	0.32 ± 0.01 (10.8)	0.10 ±0.00 (3.2)	0.01 ± 0.00 (0.3)	2.97 ± 0.06
		3	0.28 ± 0.01 (11.9)	1.47 ± 0.04 (62.0)	0.28 ± 0.01 (12.0)	0.25 ± 0.01 (10.4)	0.08 ± 0.00 (3.4)	0.01 ± 0.00 (0.3)	2.37 ± 0.06
***OsCCD4b*_Ri** x *stPAC*	**21** x 2-3-21	N	0.29 ± 0.00 (12.0)	1.45 ± 0.01 (59.8)	0.34 ± 0.01 (14.1)	0.25 ± 0.01 (10.5)	0.08 ± 0.00 (3.2)	0.01 ± 0.00 (0.4)	2.42 ± 0.01
	**21** x 2-3-16-	3	0.19 ± 0.00 (13.5)	0.76 ± 0.01 (52.9)	0.26 ± 0.00 (17.9)	0.16 ± 0.00 (11.1)	0.06 ±0.00 (4.1)	0.01 ± 0.00 (0.6)	1.43 ± 0.01
		5	0.23 ± 0.00 (13.8)	0.90 ± 0.02 (54.1)	0.27 ± 0.00 (16.2)	0.18 ± 0.00 (11.0)	0.07 ± 0.00 (4.5)	0.01 ± 0.00 (0.6)	1.66 ± 0.03
	**31** x 2-10-25	N	0.22 ± 0.00 (11.8)	1.06 ± 0.03 (55.8)	0.28 ± 0.01 (14.7)	0.24 ± 0.00 (12.7)	0.09 ± 0.00 (4.5)	0.01 ± 0.00 (0.5)	1.90 ± 0.04
	**31** x 2-10-20-	2	0.14 ± 0.01 (8.4)	1.03 ± 0.07 (62.9)	0.22 ± 0.01 (13.3)	0.19 ± 0.01 (11.5)	0.06 ± 0.00 (3.4)	0.01 ± 0.00 (0.5)	1.63 ± 0.10
		7	0.20 ± 0.01 (10.5)	1.09 ± 0.05 (56.3)	0.32 ± 0.01 (16.2)	0.24 ±0.01 (12.2)	0.08 ± 0.00 (4.2)	0.01 ± 0.00 (0.6)	1.94 ± 0.08

Data are expressed as mean (µg/g dry weight) ± SD from three independent experiments using the mature seeds of a homozygous F_4 generation that was developed from crossbreeding two independent lines for *OsCCD1*_Ri, *OsCCD4a*_Ri, and *OsCCD4b*_Ri and *stPAC 25* rice displaying carotenoid-accumulating golden color rice (Jeong et al. 2017). NT represents non-transgenic rice seeds (*Oryza sativa* L. cv. Ilmi), and N represents nullizygous rice seeds containing only an *stPAC* gene without an RNAi gene for *OsCCD* after segregation. All samples are unpolished rice seeds that were harvested at 40 DAF.

Supplementary Fig. S1

a

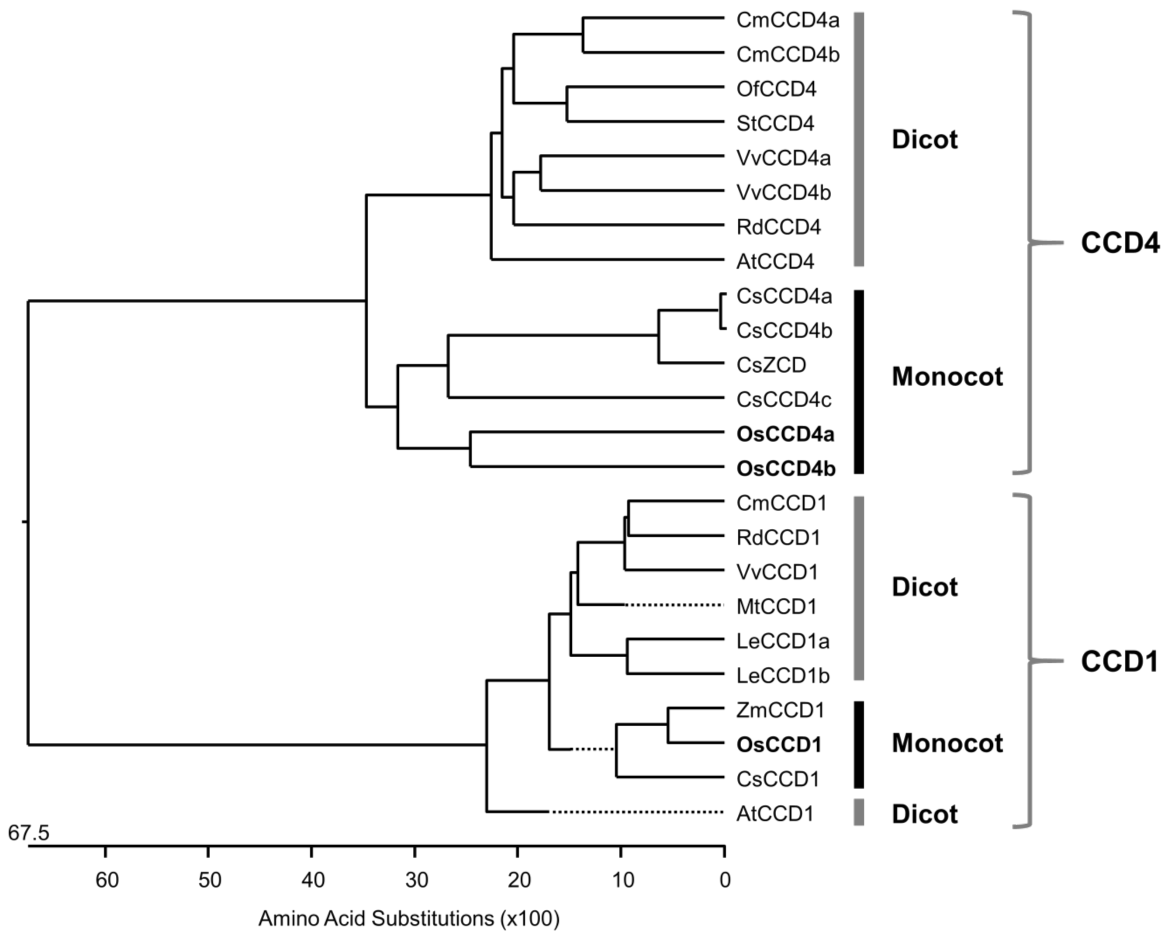

b

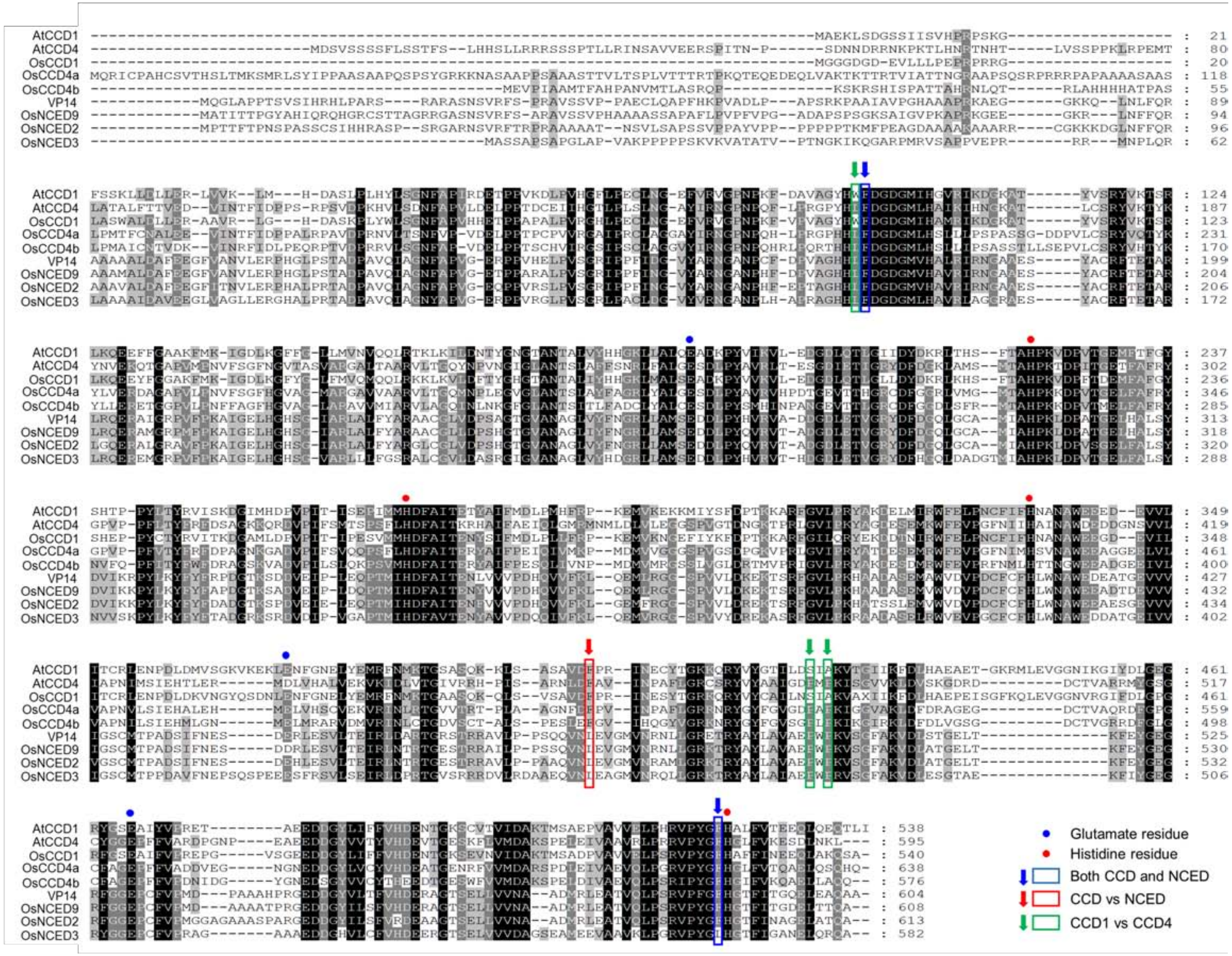
AtCCD1
AtCCD4
OsCCD1
OsCCD4a
OsCCD4b
VP14
OsNCED9
OsNCED2
OsNCED3
Glutamate residue
Histidine residue
Both CCD and NCED
CCD vs NCED
CCD1 vs CCD4

Supplementary Fig. S1. (a) Phylogenetic tree of plant CCDs on the basis of amino acid sequence similarities. The protein sequences were clustered using ClustalW algorithm. The accession numbers used here are as follows: AtCCD1 (At3g63520) and AtCCD4 (At4g19170) from *Arabidopsis thaliana*; CmCCD4a (AB247158.1) and CmCCD4b (AB247160) from *Chrysanthemum* x *morifolium*; CmCCD1 (DQ269467) from *Cucumis melo*; CsCCD1 (CAC79592.1), CsCCD4a (ACD62476.1), CsCCD4b (ACD62477.1), CsCCD4c (JN131499) and CsZCD (CAD33262.1) from *Crocus sativus*; LeCCD1a (AY576001) and LeCCD1b (AY576002) from *Solanum lycopersicum*; MtCCD1 (FM204879) from *Medicago truncatula*; OfCCD4 (ABY60887) from *Osmanathus fragrans*; OsCCD1 (Os12g0640600), OsCCD4a (Os02g0704000) and OsCCD4b (Os12g0435200) from *Oryza sativa*; RdCCD1 (ABY47994) and RdCCD4 (ABY60886) from *Rosa damascene*; StCCD4 (XP_006359966) from *Solanum tuberosum*; VvCCD1 (AY856353), VvCCD4a (AGT63321.1) and VvCCD4b (AGT63322.1) from *Vitis vinifera*; ZmCCD1 (DQ539625) from *Zea may*. (b) Alignment of protein sequences among plant CCDs including AtCCD1 and AtCCD4 from *Arabidopsis*; OsCCD1, OsCCD4a, and OsCCD4b from *Oryza sativa*; VP14 (NP_001105902) from *Zea may*; and OsNCED9 (Os03g0645900), OsNCED2 (Os12g0617400), and OsNCED3 (Os07g0154100) from *Oryza sativa* using the ClustalW algorithm. Blue dots indicate the glutamate residues associated with fixing the iron atom to ligate with a histidine residue, and red dots indicate the histidine residues directly ligating with the Fe^{2+}. The blue, red, and green boxes with the same colored arrows show the conserved residues in all CCDs and distinguishable residues between CCD and NCED groups and between CCD1 and CCD4 subgroups, respectively.

Supplementary Fig. S2

a

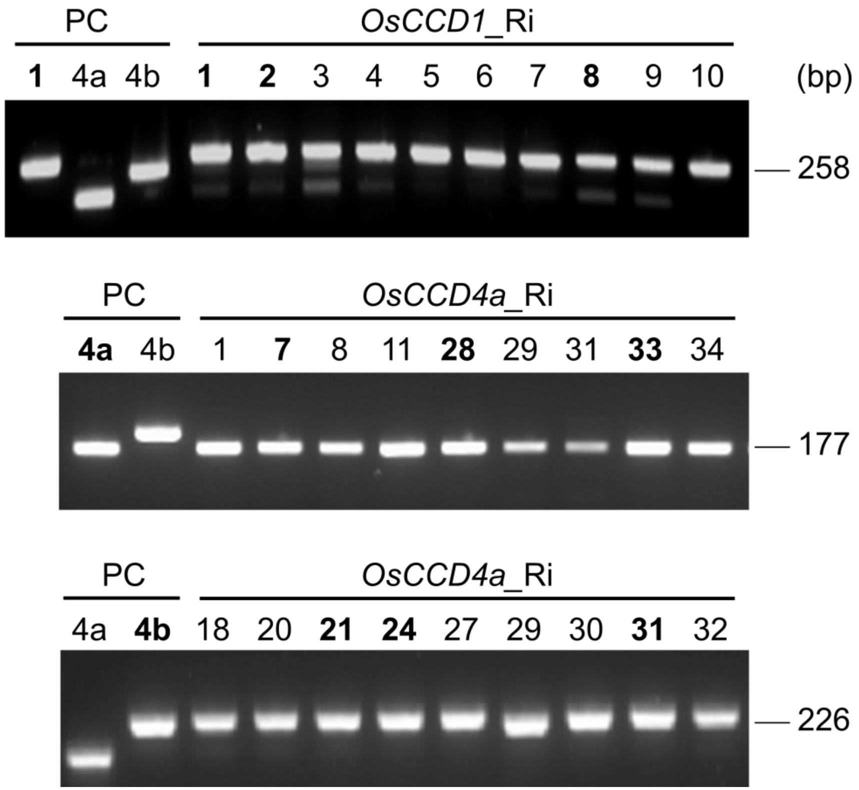

b

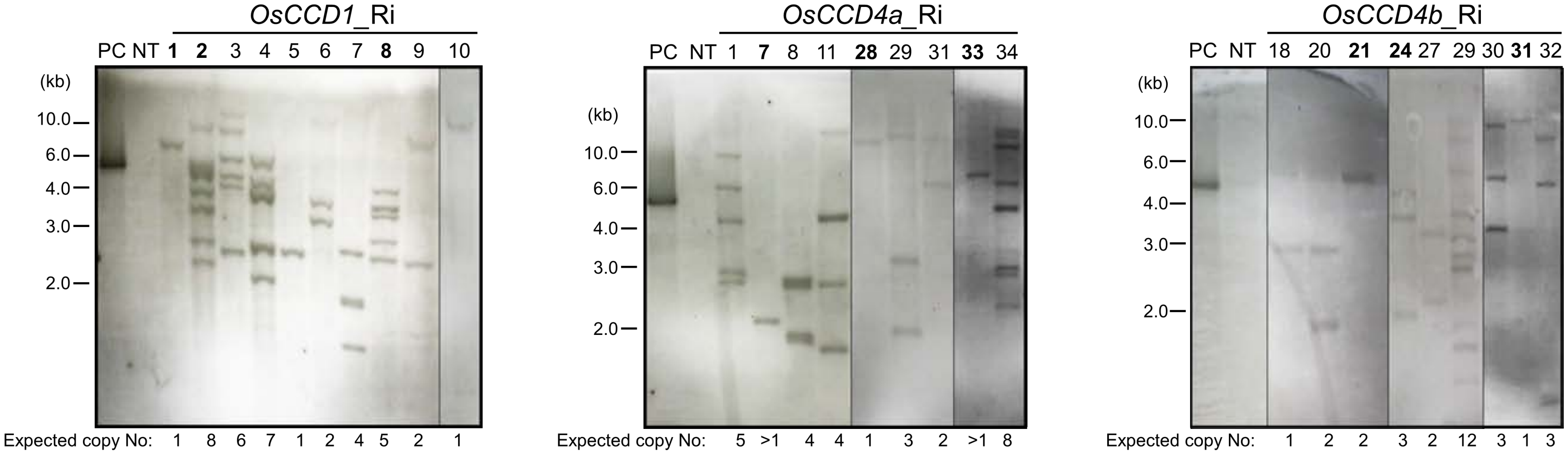

Supplementary Fig. S2. Verification of transgenic rice lines of *OsCCD1*_Ri, *OsCCD4a*_Ri, and *OsCCD4b*_Ri. (a) Genomic DNA PCR to confirm transgene insertion with leaf tissues of the T_0 generation. The expected sizes of amplicons are indicated on the right. The primer information is shown in Supplementary Table S1. (b) Genomic Southern blot analysis to determine transgene copy numbers with an NPT II probe. The DNA molecular size markers are indicated on the left. PC represents plasmid DNA of a binary vector to suppress one of the *OsCCD* genes as a positive control. NT represents non-transgenic rice plants (*Oryza sativa* L. cv. Ilmi). Line numbers for further studies are indicated in bold. The blots were rearranged to make composite figures.

Supplementary Fig. S3

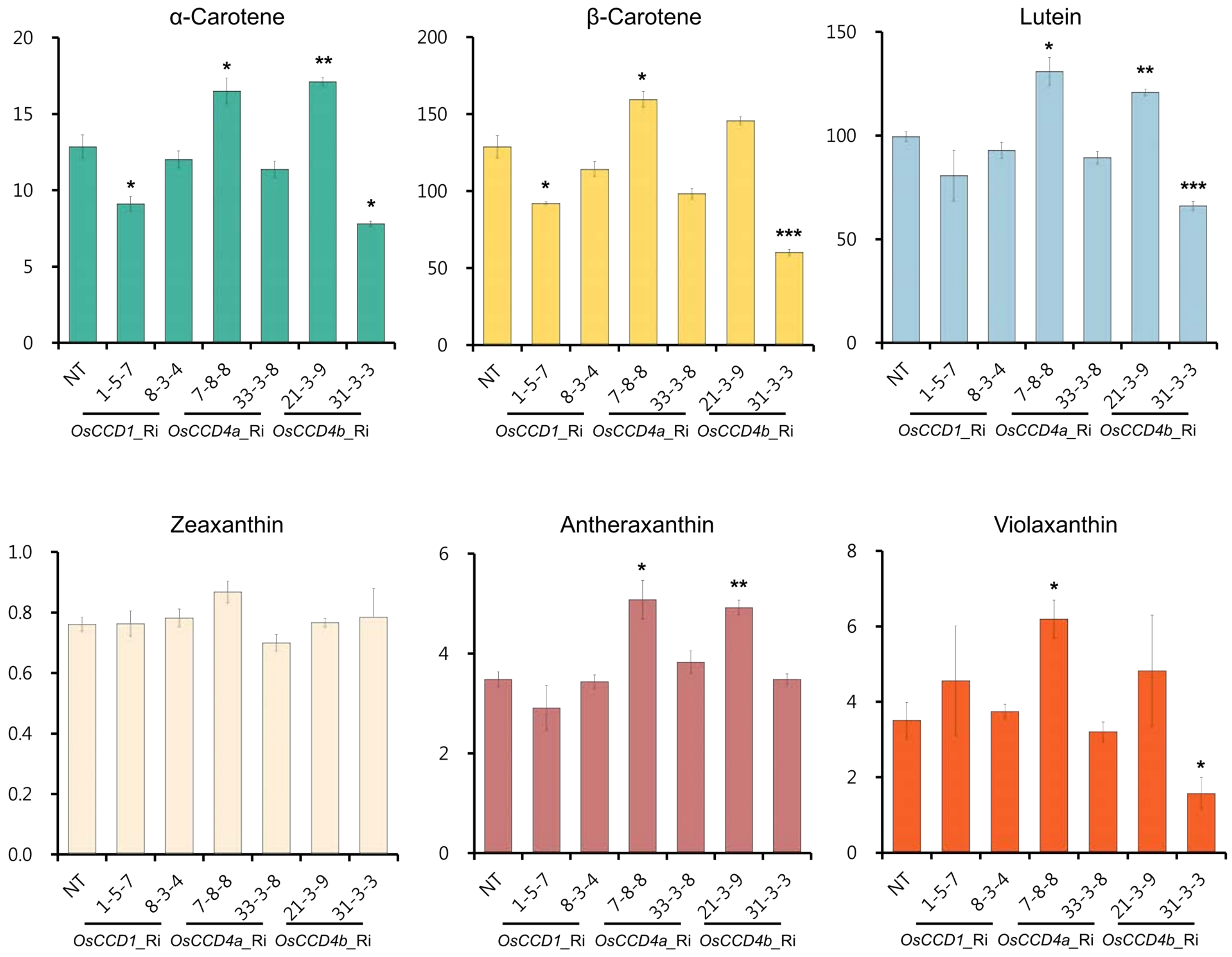

Supplementary Fig. S3. Contents of individual carotenoid components in transgenic rice leaf tissues of *OsCCD1*_Ri, *OsCCD4a*_Ri, and *OsCCD4b*_Ri. Two independent transgenic plants for each construct were analyzed with T_2 leaf tissues by HPLC. NT represents non-transgenic rice plants (*Oryza sativa* L. cv. Ilmi). Data are expressed as mean (μg/g dry weight) ± SD from three independent experiments with error bars. The relative differences to NT plants were determined using a one-tailed Student's *t*-test, and the significance of the differences is indicated as *p* values (***$p < 0.001$, ** $p < 0.01$, * $p < 0.05$).

Supplementary Fig. S4

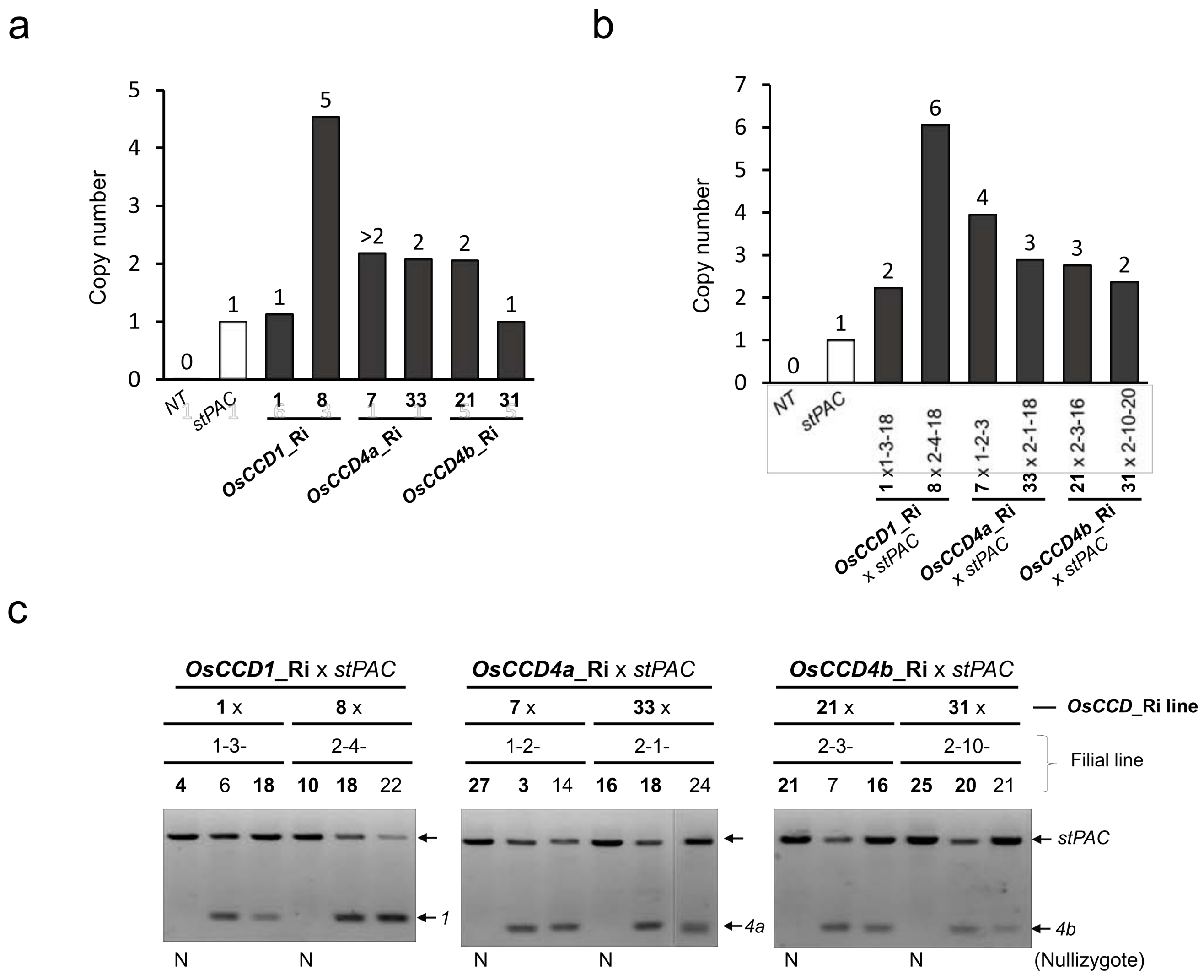

Supplementary Fig. S4. Verification of transgene homozygosity in interbred rice lines between each of two independent lines for *OsCCD1*_Ri*,* *OsCCD4a*_Ri and *OsCCD4b*_Ri (female parent), and a *stPAC* line (male parent). (a) TaqMan-PCR to confirm T-DNA copy numbers of both parents of the T_3 plant generation with a *Bar* assay. (b) TaqMan-PCR to confirm the homozygosity of two transgenes from both parents in the stacked filial lines of the F_3 plant generation after cross-fertilization and further development into next generation with a *Bar* assay. The expected copy numbers are indicated at the top of the bar in (a) and (b). (c) Genomic DNA-PCR to confirm whether the transgene is present or absent in the filial sub-lines of F_3 leaf tissues with the *attB* primer set shown in Supplementary Table S1. Number of lines in bold were used for producing F_4 seeds for carotenoid analysis by HPLC and endosperm color inspection as shown in Fig. 5 and Supplementary Table S3. NT represents non-transgenic rice plants (*Oryza sativa* L. cv. Ilmi). N represents nullizygous rice line containing only a *stPAC* gene without the RNAi gene for *OsCCDs* after segregation. One of gels was constructed as a composite figure to erase a blank space.

Supplementary Fig. S5

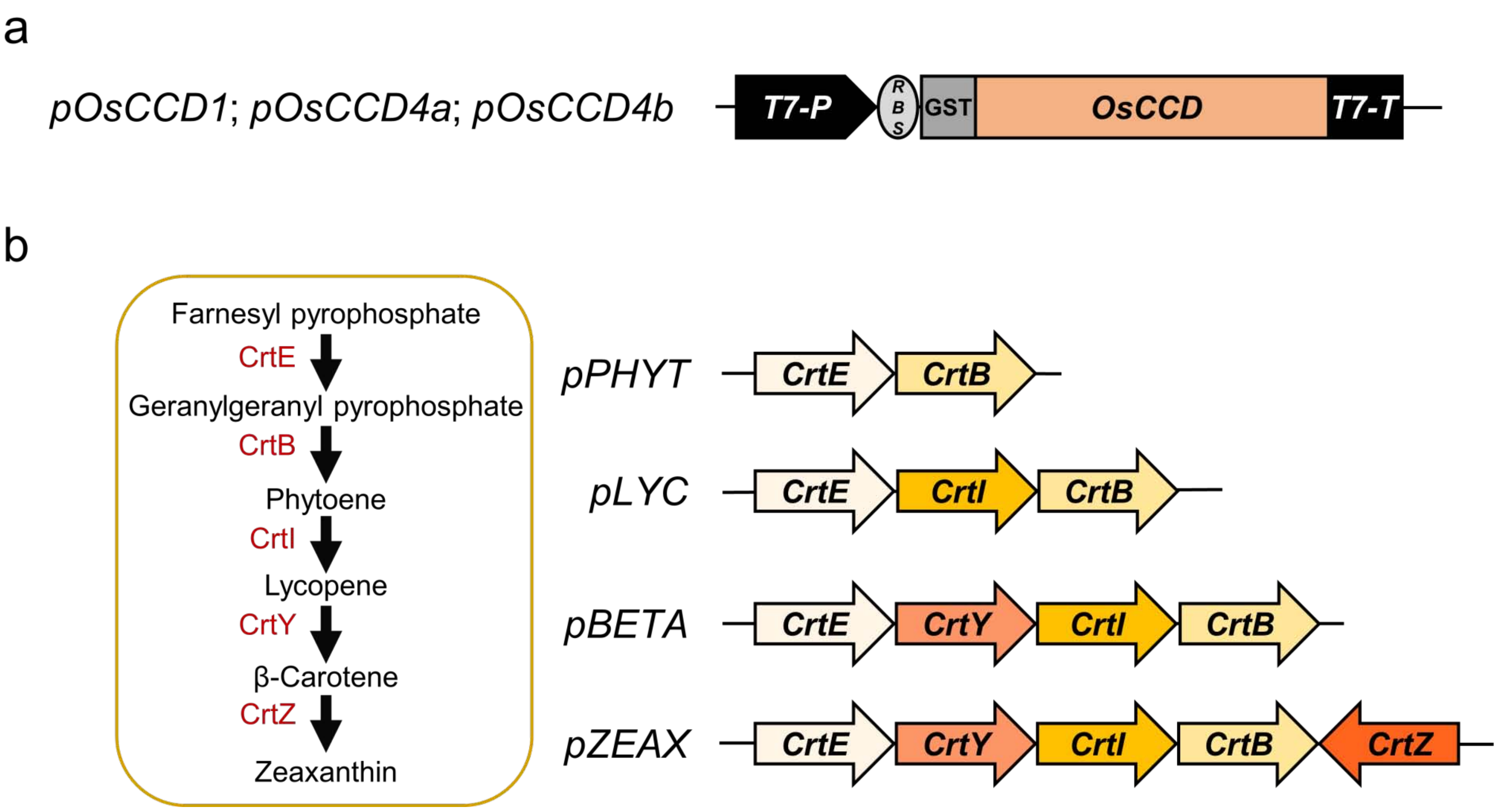

c

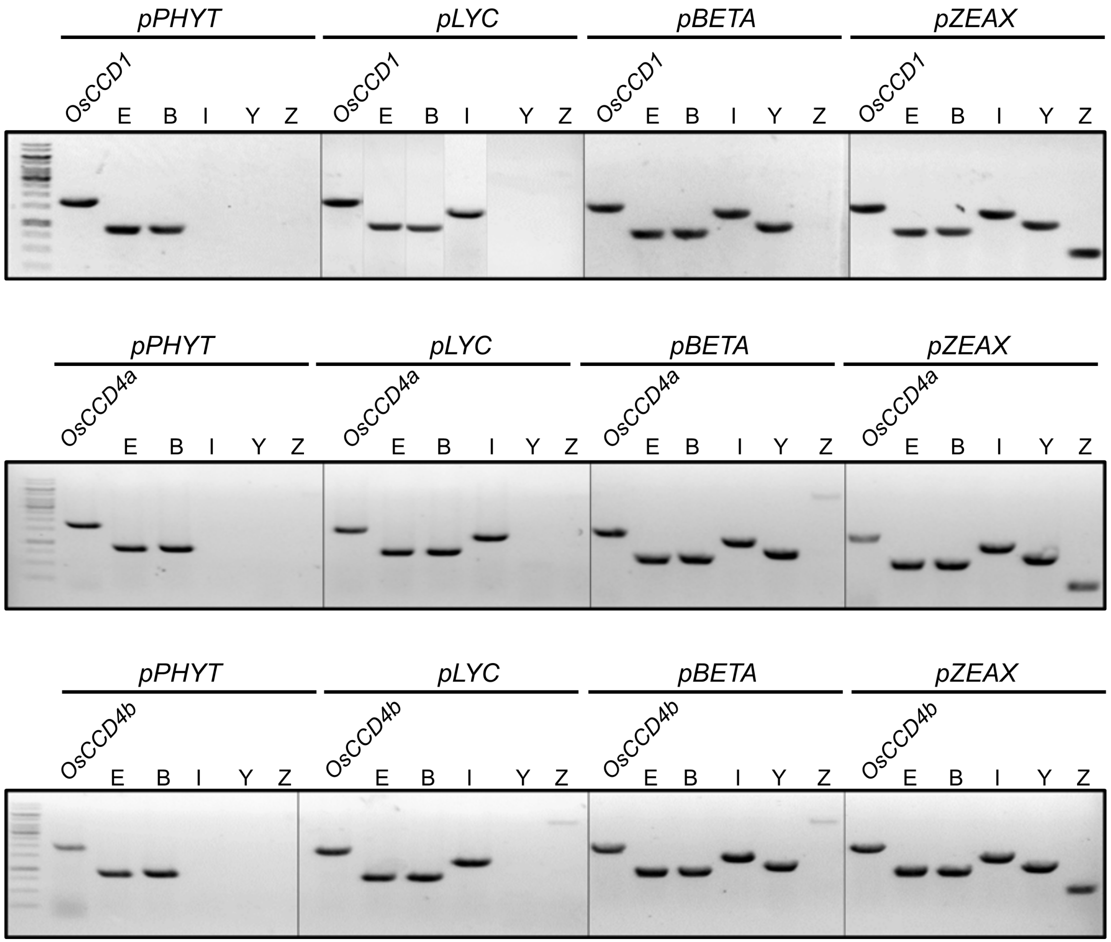

Supplementary Fig. S5. *In vitro* expression of *OsCCD 1, 4a* and *4b* genes in four carotenoid-accumulating *E. coli* strains to analyze carotenoid cleavage activities. (a) Three vectors to over-express one of the *OsCCDs* controlled by a T7 promoter (*T7-P*) and T7 terminator (*T7-T*) of bacteriophage T7 RNA polymerase. RBS and GST represent ribosomal binding sites and the N-terminally fused glutathione S-transferase gene in a *pDEST15* backbone vector, respectively. (b) Schematic diagram of enzymatic steps including five operon genes of *CrtE*, *CrtB*, *CrtI*, *CrtY,* and *CrtZ* responsible for a carotenoid locus in *E. coli* and four vectors engineered for the generation of phytoene, lycopene, β-carotene, and zeaxanthin as *pPHYT*, *pLYC*, *pBETA*, and *pZEAX* in *E. coli*. (c) Colony PCR to confirm the incorporation of *pPHYT*, *pLYC*, *pBETA*, and *pZEAX* vector in BL21-AI *E. coli* competent cells harboring one of the *pOsCCD1, pOsCCD4a*, and *pOsCCD4b* vectors. All primer sequences used here are mentioned in Supplementary Table S2. The gel images were reconstructed as composite figures with the chosen colonies being used for carotenoid analysis and color inspection in Fig. 6 among several colonies simultaneously examined.
